# In-situ laser ablation MC-ICP-MS of lead white in paintings

**DOI:** 10.1038/s40494-026-02720-3

**Published:** 2026-06-13

**Authors:** Paolo D’Imporzano, Graham A. Hagen-Peter

**Affiliations:** Department of Earth Sciences, Vrije Universiteit Amsterdam, Amsterdam, Netherlands

## Abstract

This study develops and validates an in-situ LA-MC-ICP-MS method for lead isotope ratios (LIR) measurements of lead carbonate pigments at the micrometre scale. Two matrix-matched synthetic lead whites (PDLW1 and PDLW2) were produced from metallic lead, converted to carbonate, and characterised by solution MC-ICP-MS to define reference isotope compositions. These pigments were then used as calibrants and quality-control reference materials to assess LA performance, which yields accurate isotope ratios consistent with solution data. LA analyses on samples previously analysed by solution in other studies agree with the literature data. The analysis of mock-up cross-sections shows that oil-rich, pigment-poor domains degrade signal stability and precision, whereas pigment-rich domains provide accurate LIR, proving that this approach is valid to analyse LIR of paint samples. The method successfully identified the provenance of an anonymous sample believed to be a 16–17^th^ century Italian painting. Finally, LA was used to produce isotopic maps that resolve layer-specific LIR.

## Introduction

Lead isotope ratio **(LIR)** analysis of lead white **(LW)** pigments via multi-collector inductively-coupled-plasma mass-spectrometry **(MC-ICP-MS)** is a well-established methodology for determining pigment provenance^1–7^. In addition to material sourcing, this technique successfully reconstructs historical artist movements and socio-economic trade networks, while providing critical analytical data for painting authentication and identification. However, the broader application of this technique in heritage science is currently constrained by significant methodological limitations regarding sample extraction and spatial resolution.

The primary limitation of traditional MC-ICP-MS is its reliance on invasive sampling and the need to dissolve the sample for analysis. Sampling, whether performed manually by conservators or via micro-scalpel from existing cross-sections^8^, remains fundamentally invasive for unique cultural heritage objects. Furthermore, these physical extraction methods suffer from extreme material inefficiency. While a high-precision LIR analysis requires only 50–100 nanograms of lead (ng = 10^−^^9^ g, which is one billionth of a gram, lead constitutes approximately $70 \% \mathrm{w}/\mathrm{w}$ of lead white), manual and micro-scalpel methods inevitably extract thousands of nanograms. Consequently, more than 90% of the extracted material is wasted during dissolution, as mechanically isolating exactly $100\mathrm{ng}$ of sample is physically unfeasible.

A secondary critical limitation is the inadequate spatial resolution inherent in physical sampling. When paint fragments are acquired via manual scraping, the stratigraphic integrity of the painting is lost. If the extracted fragment contains multiple paint layers, containing different historical sources of lead white, the resulting LIR data will merely reflect a homogenised, weighted average rather than a layer-specific isotopic signature. While targeted micro-scalpel sampling from embedded cross-sections improves stratigraphic control, it is still severely limited by the physical dimensions of the tools^8^. Because micro-scalpel blades measure in the tens of micrometres (µm), they frequently exceed the thickness of individual historical paint layers. Consequently, successfully isolating a pure lead white layer without adjacent cross-contamination is difficult and remains heavily dependent on the specific rheological and elasticity/brittleness properties of the aged paint.

This study proposes an alternative path to study LIR in lead white samples via MC-ICP-MS using in-situ **laser ablation (LA)**. The use of LA to study LIR in lead white samples provides certain benefits compared to classic sampling techniques: it has extremely high spatial resolution, with the laser beam diameter as small as 1 µm, and adjustable repetition rate and fluence (energy per unit area) to sample only the needed material for LIR, without wasting sample material. This resolution also provides the possibility to sample single particles of lead white in a sample, allowing the comparison of different lead white in painting, as in the case of LW present in ground, prime, and highlights, to detect precisely retouching, reuse of canvas, and other artist practices. The samples can be, similarly to the use of micro-scalpel^8^, sampled from cross-sections, avoiding the necessity of taking new samples from paintings if not needed. The sample can be analysed without sample preparation, reducing analysis time.

LA of lead white, a lead carbonate that is applied mainly in linseed oil in oil painting, presents specific analytical challenges that must be addressed for reliable heritage science applications. LA-MC-ICP-MS inherently yields lower precision (higher external error, $2\mathrm{SD}$) compared to traditional solution-based analysis due to transient signals. This reduced precision can directly limit the ability to resolve minute isotopic differences crucial for determining pigment provenance. To be a viable alternative for painting characterisation, LA analysis must demonstrate sufficient resolving power. For example, distinguishing Venetian from 17^th^ century Dutch lead white requires resolving a difference of approximately 2700 ppm in the ^206^Pb/^204^Pb ratio^1^. Because solution-based LIR analysis achieves a 2 SD of roughly 150 ppm, easily resolving this geographic variation, LA methodologies must be optimised to approach this level of precision.

Furthermore, the physical and chemical properties of the carbonate in the paint matrix complicate the ablation process. Among others, these so-called “matrix-effects” may include (1) laser-induced elemental and isotopic fractionation during ablation and aerosol transport due to phase changes during the ablation process; (2) sample-dependent differences in isotopic fractionation in the MC-ICP-MS due to differences in sample load (“plasma loading”); (3) variable matrix-based interferences, for example, organic complexes originating from the organic matrix of the pigment (e.g., linseed oil)^9–13^.

Finally, validating the accuracy of LA-MC-ICP-MS is currently constrained by the absence of characterized, matrix-matched isotopic reference materials for lead carbonate or historical lead white pigments.

This work addresses these limitations by developing and validating an in-situ LA-MC-ICP-MS approach for LIR analysis of lead white in paint samples. Two lead white reference materials (**PDLW1** and **PDLW2**) were synthesised from metallic lead and converted to carbonates; their isotopic compositions were first established by solution MC-ICP-MS and subsequently used as calibrants and quality-control reference materials for LA measurements. These materials were employed to evaluate LA performance on lead carbonates, including signal stability and mass-dependent fractionation, and to assess their suitability as matrix-matched reference materials.

To benchmark the proposed approach against established datasets, the 16th-century Venetian lead white samples previously reported in D’imporzano et al. ^1^ were re-analysed in this study via solution and by LA and directly compared with literature data^1^. In addition, a stratified mock-up cross-section was prepared consisting of two discrete layers of PDLW1- and PDLW2-based oil paint, separated by zinc white interlayers, to reproduce a realistic multilayer paint stratigraphy. This mock-up was analysed to quantify isotopic fractionation in an oil-bound carbonate matrix and to test the spatial resolving power of LA via isotopic mapping, i.e., the ability to discriminate isotopically distinct lead white domains within a single cross-section.

Finally, the validated method was applied to an anonymous paint sample dated to the 16th–17^th^ century, presumably produced in Italy, to identify the isotopic signature of the lead source used in the lead white pigment.

## Method

### Lead white

**Fig. 1:**
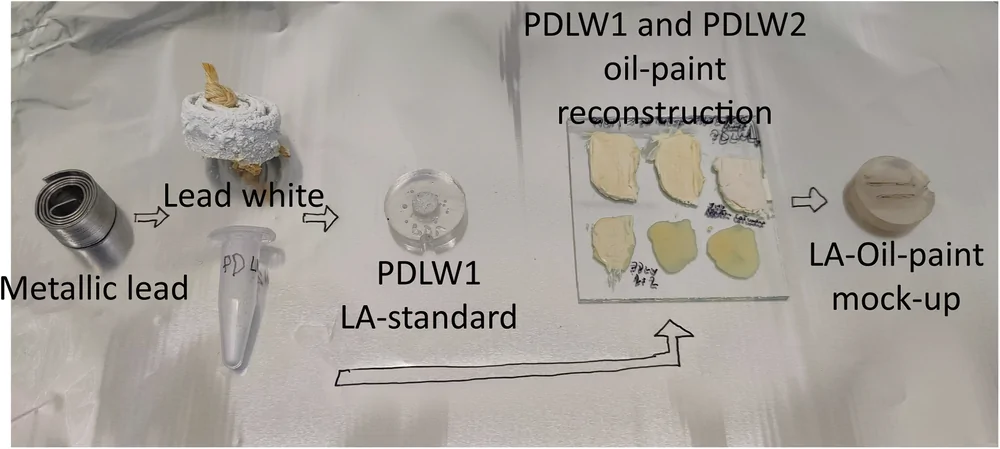
Photographs of the PDLW1 reference material at successive preparation stages (left to right): metallic lead precursor, synthesised lead white pigment, epoxy-embedded pellet for use as an LA standard, and lead white dispersed in linseed oil applied on glass to produce a mock-up paint sample.

The lead white pigments used in this study, PDLW1 and PDLW2, were synthesized from metallic lead fishing weights purchased online, manufactured in China. The lead was supplied as small coils approximately 2 cm in height (Fig. 1, *Metallic lead*). These coils were first analysed to determine their Pb isotopic composition and to assess isotopic homogeneity within each coil; the metal was found to be isotopically homogeneous within individual coils, and two coils with distinct isotopic signatures were selected to produce lead whites with different isotopic compositions (PDLW1 and PDLW2, see S1 supplementary material Supplementary Note 1*: metal LIR vs LW LIR*). To synthesise lead white, the selected coils were suspended above acetic acid at room temperature for two weeks to form lead acetate. The coils were then put above a solution of water, sugar, and baking yeast, which generated CO_2_ and promoted the conversion of lead acetate to lead carbonate over a further two weeks. The lead white that formed on the surface of the coils was subsequently removed, thoroughly washed, and homogenised (Fig. 1, *Lead white*).

PDLW1 was used as the bracketing standard and PDLW2 as the quality-control material for the laser ablation measurements; this assignment of roles between the two pigments was arbitrary (they could, in principle, be swapped), whereas the non-arbitrary decision was to use our own synthetically prepared lead-white carbonate as the reference matrix to mitigate matrix effects. Lead-carbonate pigment was considered to be more appropriate for **Standard Sample Bracketing (SSB)** in LA-MC-ICP-MS work than a non–matrix-matched silicate glass such as NBS/NIST glasses, because of the very large differences in Pb and trace-element concentrations between glass and lead carbonate (often several orders of magnitude; e.g., NIST 610 with ~430 ug/g Pb and lead white with 70-80 wt% Pb). Significantly different Pb signals resulting from the ablation of Pb white vs glass, or significantly different fluxes of total material into the plasma if the former is compensated by using a larger spot size for the glasses, can lead to differences in the magnitude of instrumental mass-bias between glasses and Pb white. Moreover, using a synthetic Pb carbonate standard better captures the specific ablation behaviour, particle generation, and signal instability associated with the surface structure and crystallographic structure of carbonates and phase differences (e.g. ratios between cerussite/hydrocerussite, presence of partial unreacted lead product as acetates, plumbonacrite, metallic lead and traces of galena)^14–18^.

The lead white thus formed was used to test the LA method, together with a third type of lead white, which was already analysed in D’Imporzano et al. 2021^1^, coming from historical Venetian lead white, and here named **GN**. The lead white pigments were then prepared in different forms for the analysis as listed below:

Pure pigment powder for solution analysis; The powder of PDLW1, PDLW2, and GN was dissolved in HNO_3_ and used to analyse the pigments in solution.

Epoxy-embedded (binder-free) synthetic lead white pigments (PDLW1 and PDLW2); The two lead white pigments were embedded in epoxy resin for use as LA standards and quality-control reference materials. These binder-free pellets serve as the primary calibration materials throughout this study.

Epoxy-embedded (binder-free) historical lead white (GN); Pigment powder from GN was embedded in epoxy resin and re-analysed in this study to provide an independent benchmark for assessing the accuracy and precision of the LA method against an established literature dataset.

Mock-up cross-sections containing PDLW1 and PDLW2 in oil-paint layers; A stratified paint mock-up was prepared by applying alternating layers of PDLW1 and PDLW2 dispersed in linseed oil, separated by zinc white interlayers, on a glass support. The mock-up was artificially aged and embedded to produce cross-sections representative of a multilayer paint stratigraphy. These samples were used to assess isotopic fractionation in an oil-bound carbonate matrix and to evaluate the spatial resolving power of LA through isotopic mapping.

Anonymous paint sample (16–17^th^ century, Italian); An embedded cross-section from an anonymous painting, believed to have been produced in Italy during the 16th–17^th^ century, was analysed to demonstrate the method’s applicability to real heritage material and to identify the provenance of the lead white pigment present in the sample.

### Instrumentation

Lead isotope ratio measurements of solutions were performed in dry plasma using a Thermo Fisher Neoma MC-ICP-MS coupled with an Apex omega desolvator (parameters in Table 1). Ion beams corresponding to the following isotopes were measured on Faraday cups coupled to 10^11^-ohm amplifiers: ^200^Hg, ^202^Hg, ^203^Tl, ^204^(Hg, Pb), ^205^Tl, ^206^Pb, ^207^Pb, ^208^Pb, ^209^Bi. The samples for solution works were introduced as solutions of 25 ppb lead doped with 5 ppb of Thallium (2 mL for each sample was prepared). The Thallium was used to calculate the mass-bias fractionation factor β using the exponential law internally during each measurement. The mercury Isotope ^202^Hg was monitored and used to subtract the isobaric interference (isotope ^204^Hg) from the lead isotope ^204^Pb. For each run, an in-house quality control standard (“CPI”) and blanks were also analysed. In solution work, blanks were below 50 pg, and therefore their contribution to the analysis is considered irrelevant. Replicate measurements of the CPI in-house standard have been used to calculate the external precision of the Neoma in this work.

**Table 1:** Operating conditions used for the LA-MC-ICPMS measurements

Laser ablation	Settings 1	Settings 2
*He (L min*^-1^*)*	0.980–01.080	0.980–01.080
*speed µm/s*	1	2
*spot µm*	5	5
*Energy (J)*	0.42	0.51
*Hz*	7	8
*N (dosage)*	35	20
N*Energy	10.2	14.7
*Neoma Integration time (s)*	0.5	0.25
*Ablation time sec*	~50	~20
*Ion Yield %*	0.05–0.3	0.05–0.3
**Plasma**	**Solution**	**Laser**
*RF Power (W)*	1200	1200
*Plasma condition*	dry plasma	
*Nebulizer Ar flow (L min*^−*1*^*)*	0.8	
*Sampling cone*	Standard	Standard
*Skimmer cone*	H	H
**Collection**	**Solution**	**Laser**
*Sensitivity (V ppm*^*-1*^*)*	615–770	
*Blank signal* ^*208*^*Pb (V)*	<0.003	<0.001
*Cycles*	40	~80–100
*block*	3	1
*Integration time (seconds)*	4	0.25–0.5

The table summarises the principal laser ablation parameters (spot size, repetition rate, scan speed, carrier gas configuration etc.) and the corresponding MC-ICPMS acquisition settings (cup configuration, integration time, mass etc.) applied for lead isotope ratio analysis of lead white.

For laser ablation analysis, the ESL (Elemental Scientific Lasers) imageGEO193 193 nm ArF excimer laser ablation system fitted with a TV3 ablation chamber was used. Ablations were carried out in a 2-volume chamber in a He atmosphere, and the ablated aerosol was mixed with Ar in a signal-smoothing bulb before introduction into the plasma. The analysis parameters for the Neoma and the LA are reported in Table 1. The PDLW1 was used as a primary standard to correct for instrumental mass bias via a SSB correction. A 70 and 60 s pause was applied before and after each ablation, respectively, to reduce any memory effect (background signal on the 208Pb < 0.001 V). The data were reduced using Iolite 4, via the DRS Pb Isotopes DRS (Beta). For each run, samples were corrected for the baseline (calculated via a spline function), collecting the background signal for 30 seconds before each sample, and the isobaric interference on ^204^Pb was corrected for ^204^Hg using the ^202^Hg.

Laser ablation mapping was performed using different parameters obtained using a dedicated Iolite software provided by ESL laser (iolite_optimizer_v0.96). A specially designed ESL plasma torch was used to improve the washout time. The imaging was performed with a raster using a 3 µm spot size (0.5 µm separation between lines to avoid resampling), 20 Hz repetition rate, and 20 µm/s scan speed over a 1 mm × 0.3 mm area.

### Software and data

The Neoma data were collected using the dedicated Thermo Fisher Neoma software Qtegra. The LA data are reduced using the Software Iolite 4, using the DRS Pb Isotopes DRS (Beta). The statistical analysis is done in RStudio using the package ggplot2 (Rcodes zip files in Supplementary material). The data produced in this study are reported in the supplementary material as an Excel file, Data S1 and Data S2.

## Results

### Solution analyses

Table 2 reports the external precision as 2 SD for the in-house standard solution, CPI, analysed during the course of this project (6 months c.a. for a total of 203 replicates of the solution) in dry plasma. The data are within error with literature data for the same sample analysed in^1,2,4,7,19–22^ (which reports much data on lead isotopes of lead white and other medium as silver and lead glaze). A more detailed discussion on the comparison of this data with literature data is given in the S1 Supplementary material Supplementary Note 2: S*hort discussion on the comparison with the Neptune*.

**Table 2:** Results of the analysis of CPI in-house standard over a period of 6 months, *n* = 203

		²⁰⁶Pb/²⁰⁴Pb	2 SD	²⁰⁷Pb/²⁰⁴Pb	2 SD	²⁰⁸Pb/²⁰⁴Pb	2 SD	²⁰⁸Pb/²⁰⁶Pb	2 SD	²⁰⁷Pb/²⁰⁶Pb	2 SD
**Neoma** *n* = **203**	CPI in-house Tl	17.9006	0.0015	15.5665	0.0020	38.0230	0.0060	2.12409	0.00018	0.86961	0.00005

Comparable external precision was achieved for analysis of the solution of the metal and the pigments of lead white synthesised in this work (PDLW1 and PDLW2), as reported in detail in S1 Supplementary material section Supplementary note 1*: metal LIR vs LW LIR*.

### Comparison between LIR via laser ablation and solution analysis

Laser ablation settings were selected to minimise invasiveness while maintaining accuracy and reproducibility. Testing over 8 months established that low fluence (0.42–0.51 J/cm², corresponding to 1–5% of the system’s energy output) was sufficient to produce a strong signal (6–9 V on ²⁰⁸Pb) given the nature of carbonates. The two best-performing settings, PDLW2‑10.2 and PDLW2‑14.7 (Settings 1 and 2 in Table 1), were selected on this basis; full details of the selection process are provided in S1 Supplementary material (Sections: Supplementary note 3*: Decision on the optimal laser ablation settings for lead white and Laser ablation: raster vs line*).

Using a 5-µm-diameter circular spot, each analysis ablated either a 50-µm line or a 15×15-µm raster, removing approximately 8 ng of lead white pigment (~1.25×10⁻⁹ cm³, estimated ablation depth ~5 µm; see S1 Supplementary material paragraph Supplementary note 4*: Material ablated and efficiency*). All LA analyses used SSB correction with PDLW1 as the bracketing standard. PDLW2‑10.2 was measured across 16 replicates over 4 months; PDLW2‑14.7 across 28 replicates over the same period. For comparison, solution analyses were performed on 40 replicates over 6 months.

**Fig. 2:**
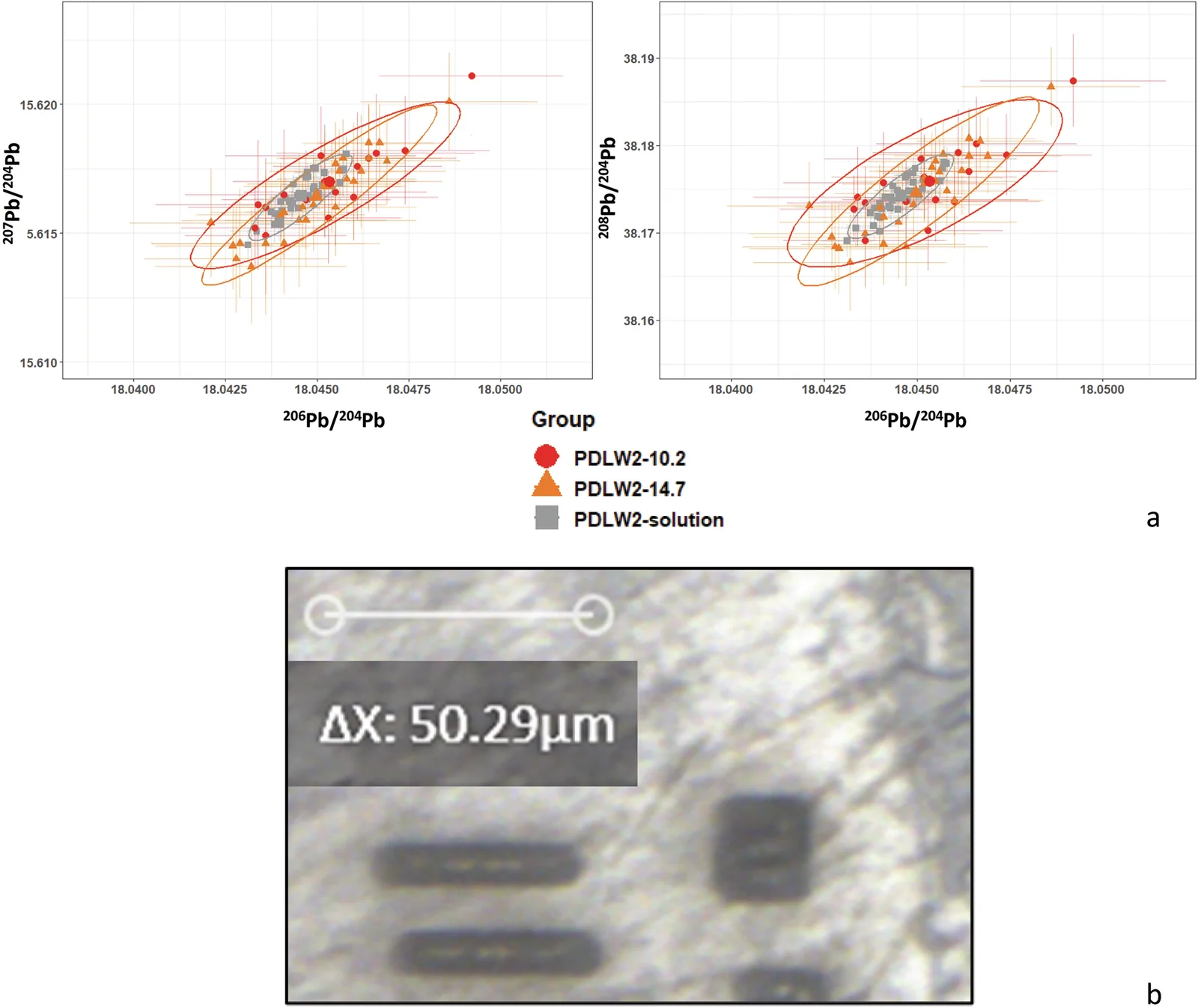
Lead isotope ratios graphs compare the data obtained via solution analysis vs laser ablation analysis. **a** Summary of laser ablation analysis vs solution analysis and corresponding analytical performance for PDLW2, mean LIR values, 2 SD, and MSWD are reported in Table 3. Ellipses represent the method-specific covariance (95%). **b** Reflected-light microscope image acquired on the LA system showing representative ablation features in the lead white pigment; the ablation depth is estimated at approximately 5 µm.

As shown in Fig. 2 and reported in Table 3, both settings yield LIR values in good agreement with solution results, and their 2 SD external precision is comparable to that reported for solution analysis of lead white in recent studies (Table S1 for CPI analyses on the Neptune^1,4–8^).

**Table 3:** Mean LIR values, 2 SD, and MSWD of the three Pb isotope ratios over ^204^Pb, obtained in this study with different settings and different matrices

		²⁰⁶Pb/²⁰⁴Pb	2 SD	MSWD	²⁰⁷Pb/²⁰⁴Pb	2 SD	MSWD	²⁰⁸Pb/²⁰⁴Pb	2 SD	MSWD
	PDLW2-10.2	18.04534	0.00322	0.46	15.61697	0.00302	0.51	38.17587	0.00881	0.78
Fig. 2	PDLW2-14.7	18.04498	0.00296	0.44	15.61642	0.00309	0.67	38.17454	0.00947	1.11
	PDLW2-solution	18.04457	0.00125	0.85	15.61644	0.00152	1.68	38.17422	0.00433	1.8
	GN	18.37918	0.00172	1.63	15.67607	0.00191	2.49	38.53978	0.00562	2.8
	GN 10.2	18.38195	0.00526	0.72	15.67863	0.0068	1.3	38.54767	0.02076	2.34
Fig. 3	GN 14.7	18.38083	0.00508	0.85	15.67782	0.00514	1.21	38.5458	0.01512	1.74
	GN D’ Imporzano et al. 2021	18.37881	0.00276	15.23	15.6754	0.00308	7.58	38.53711	0.01088	45.43
	PDLW2-solution	18.04457	0.00125	0.85	15.61644	0.00152	1.68	38.17422	0.00433	1.8
Fig. 4a	PDLW2 long	18.04498	0.00296	0.44	15.61642	0.00309	0.67	38.17454	0.00947	1.11
	PDLW short	18.04568	0.0042	0.2	15.61761	0.00403	0.25	38.17746	0.00999	0.27
	GN-solution	18.37918	0.00172	1.63	15.67607	0.00191	2.49	38.53978	0.00562	2.8
Fig. 4b	GN long	18.38083	0.00508	0.85	15.67782	0.00514	1.21	38.5458	0.01512	1.74
	GN short	18.38312	0.00649	0.43	15.67974	0.00634	0.5	38.55232	0.01761	0.68
Fig. 5	PDLW2-Oil	18.04386	0.00551	0.69	15.61416	0.00587	0.93	38.16603	0.01807	1.46
	PDLW2-14.7	18.04498	0.00296	0.44	15.61642	0.00309	0.67	38.17454	0.00947	1.11

The LA LIR ratios normalised to ^204^Pb yielded 2 SD values of 150–300 ppm with **mean squared weighted deviation (MSWD)** between 0.44 and 1.1, whereas solution analyses on the same samples produced smaller 2 SD of 80–150 ppm and MSWD of 0.85–1.8, Table 3. This indicates that the repeatability of laser ablation measurements is predicted by their measurement uncertainties, with no evidence for significant micro-scale heterogeneity or random laser-induced fractionation. Overall, the laser ablation data provide reproducible Pb isotope fingerprints suitable for provenance studies, with roughly twofold lower precision than solution measurements using Tl doping on the same instrument.

Taking into account that the amount of sample analysed is only 8 ng and that an average painting sample taken manually can be estimated, for most cases, to be in the range of 5 to 0.01 milligrams, means that using in-situ LA on cross-section is possible to reduce the amount of sample used by 500000 to 1000 times. This procedure therefore drastically reduces the amount of sample needed, preserving most of the sample intact for further analysis, while providing high-precision isotope data comparable with literature data.

### Historical Venetian lead white (GN)

**Fig. 3:**
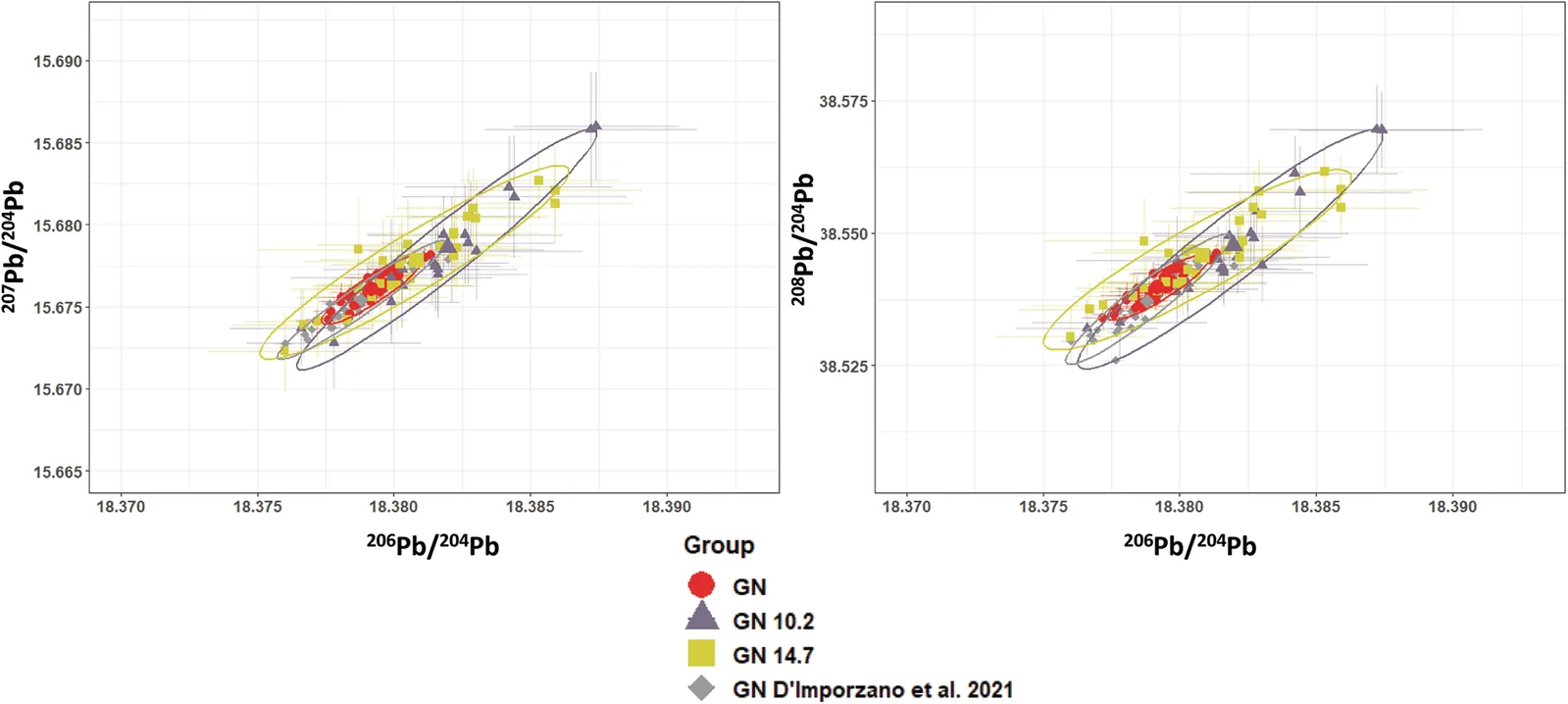
Lead isotope ratios for the historical sample GN show agreement between laser ablation, solution analysis, and published literature values. The graphs show solution analysis vs laser ablation analysis and literature data; mean LIR values, 2 SD, and MSWD are reported in Table 3. Ellipses represent the method-specific covariance (95%).

This study presents new measurements of lead white from a Venetian Adriatic shipwreck (GN), previously characterised in D’imporzano et al. (2021)^1^, and compares them with those earlier results. GN was re-analysed by solution MC-ICP-MS (Neoma) and by LA-MC-ICP-MS on a dedicated epoxy-embedded GN mount, using the same analytical protocol and LA operating conditions (14.7 and 10.2) applied to PDLW1 and PDLW2 and acquired within the same analytical session (Fig. 3, Table 3). The results show that the Neoma solution (GN Fig. 3) data are within error with the earlier measurements reported in D’ imporzano et al. (2021)^1^ (GN D’Imporzano et al. ^4^ in Fig. 3). The laser ablation data, for both LA settings (GN 10.2 and GN 14.7), are also accurate relative to the Neoma solution measurements, with GN 14.7 setting providing superior accuracy and reproducibility. However, the external error (2 SD) for the LA analyses is worse than for newly synthesised PDLW2, with 2 SD values for the three LIR being on average about 40% larger compared to the one reported in Fig. 2. This worse precision can be attributed to the history and condition of GN: the pigment remained under seawater for approximately 400 years and, when recovered, exhibited an external black alteration layer that was only partially removed in the material available for this study (no fresh sample was available). Although only visually pure white areas were targeted by laser ablation, nanoscale impurities or residual alteration products may have been present in some regions, thereby influencing the laser ablation and resulting in higher 2 SD. This could also explain why the settings 10.2 performed worse than 14.7, as this ablation is faster and can be more susceptible to small local sample phase heterogeneity, resulting in a less stable signal. ﻿Despite this, the 14.7 setting yields results that are in good agreement with the solution data and still provide sufficient resolution (approximately 270–390 ppm 2 SD for the three 204Pb-normalised LIR) for provenance studies.

### Further reducing the ablation size

One of the main aims in this study is to reduce the sample usage to a minimum. Therefore, the method was also tested on smaller areas of both PDLW2 and GN. In these tests, the laser was applied either to a raster area of approximately 10 by 10 µm or to a single line of about 20 µm in length, corresponding in both cases to a total ablated mass of roughly 3.25 ng of LW for a 20 seconds ablation time. For this test, only the setting at 14.7 was used, as otherwise the integration time of the analysis would have been too short (10.2 has a speed of 2 µm/s and the total analysis time would have been less than 10 seconds).

**Fig. 4:**
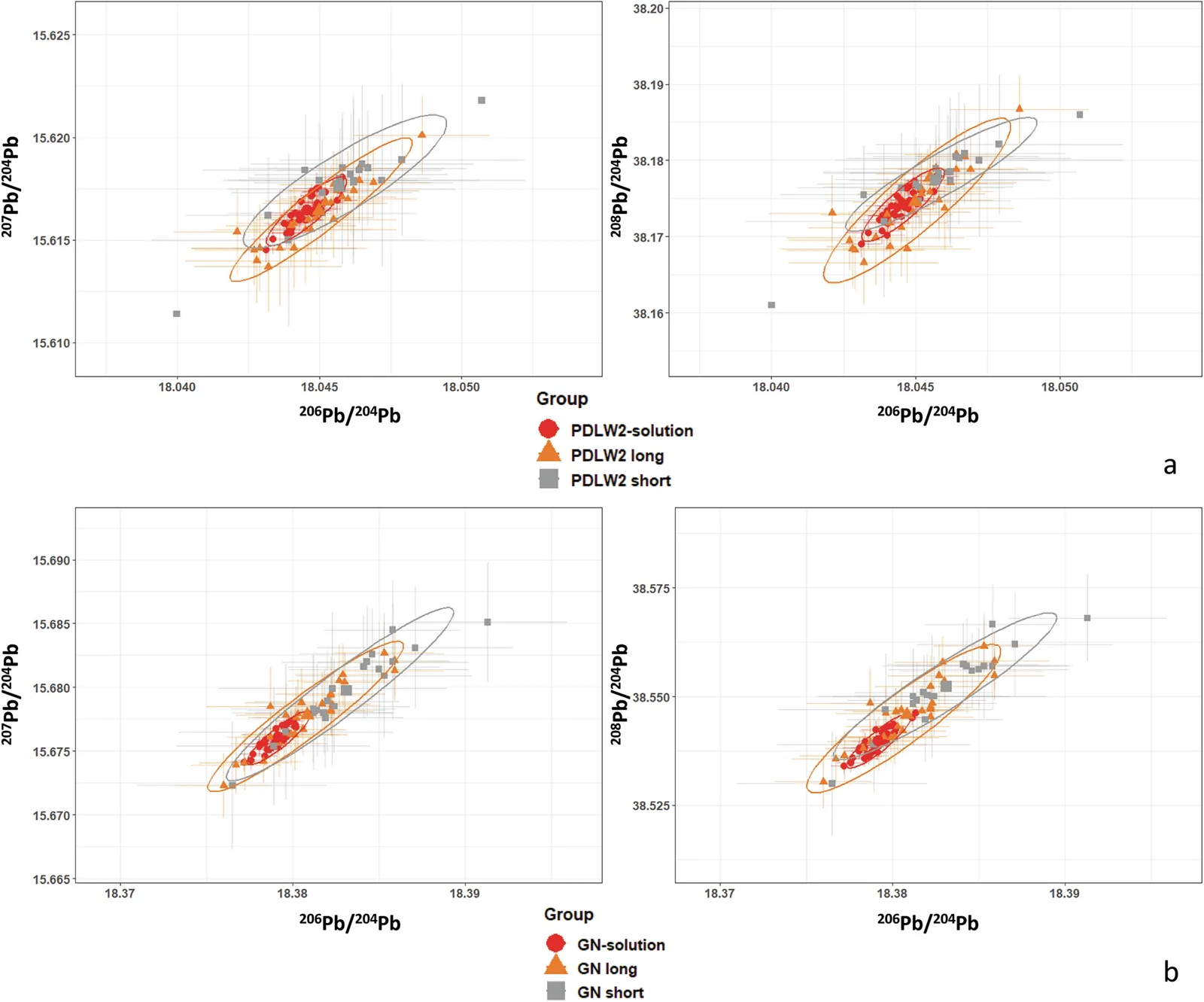
Lead isotope ratios show that ablation time (20 s vs 50 s) affects measurement accuracy relative to the time of ablation. **a** Summary of two laser operating conditions (short and long) vs solution analysis for PDLW2, mean LIR values, 2 SD, and MSWD are reported in Table 3; long corresponding to a 50 second analysis time, short is 20 second analysis. Ellipses represent the method-specific covariance (95%). **b** Summary of laser operating conditions and corresponding analytical performance for GN, which show identical patterns as discussed for PDLW2.

Consistent with previous results, PDLW2 exhibits better 2 SD values than the analysis performed on GN. As expected, the shortened analysis time for the 20 µm length measurements (PDLW2 and GN short in Fig. 4) yields a higher 2 SD compared to the longer 50 µm laser ablation method, a direct consequence of the total integration time being halved. However, while precision is reduced, the data remain accurate; this makes the method viable for identification and provenance of unique samples containing minute lead white particles, though the longer ablation protocol is recommended for optimal results when enough material is available.

### Mock-up analysis

The LA method (14.7), considered more stable for heterogeneous samples as in the case of pigment in oil, was applied to a multi-layered mock-up (Fig. 1) containing PDLW2 dispersed in linseed oil and artificially aged for three months at 40°C (each layer was applied after the layer below was hard to touch). The sample consists of four distinct layers applied on glass, alternating between Zinc Oxide, PDLW1, and PDLW2, which were subsequently embedded. The application of the LW pigments resulted in a stratigraphy that is less uniform than that found in masterworks, characterised by significant heterogeneity in particle size and varying pigment density throughout the lead white layers (Fig. 6). While this irregularity presents analytical challenges and may differ from real painting samples, it effectively simulates non-idealised conditions, offering valuable insights into Laser Ablation behaviour across a wide range of pigment-to-binder ratios and aiding in the optimisation of sampling strategies for complex real-world samples.

**Fig. 5:**
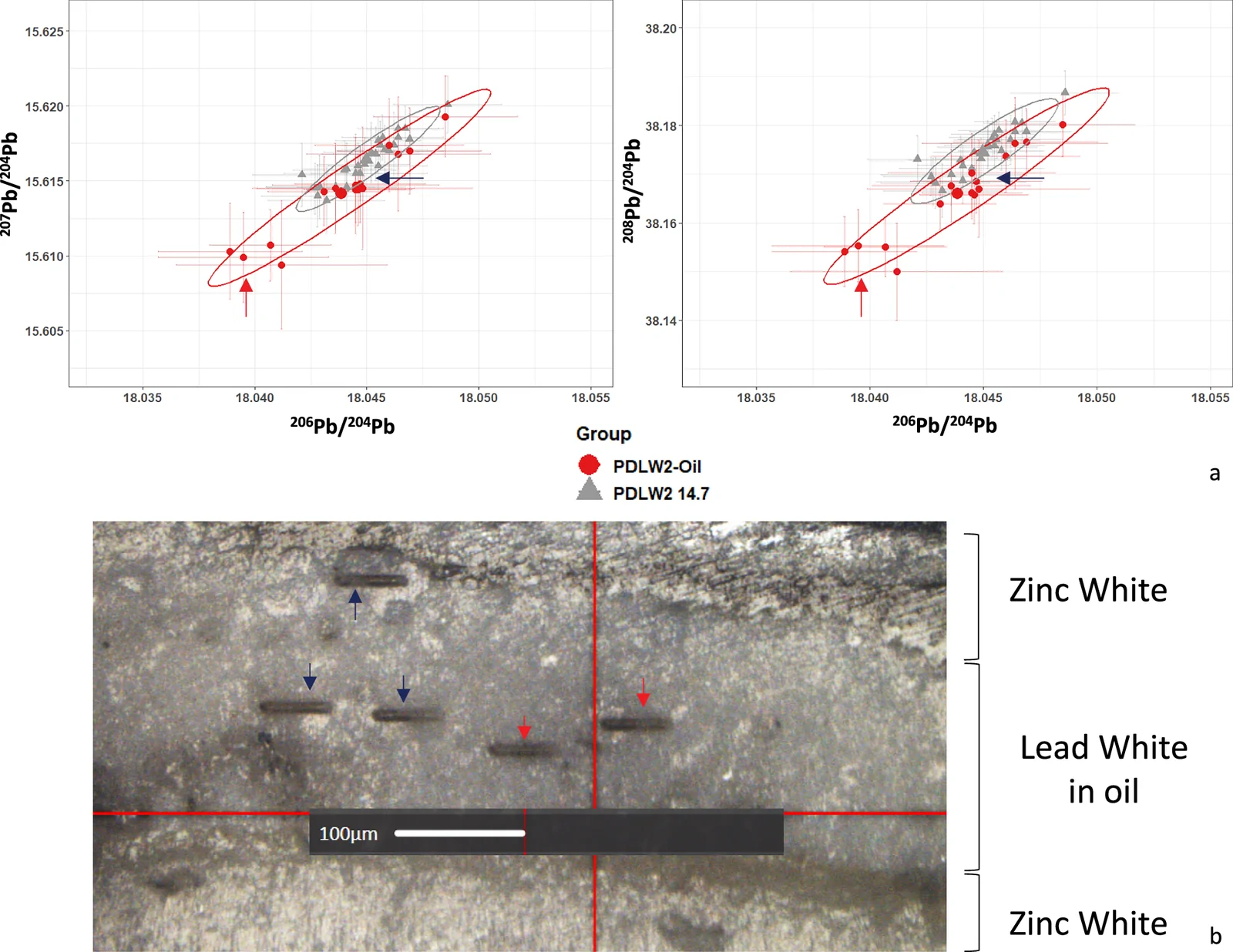
Lead isotope ratios graphs reporting the laser ablation analysis of mock-up oil paint made with PDLW2 compared with the analysis of the pure pigment. **a** Summary of laser operating conditions and corresponding analytical performance for PDLW2 in the mock-up and embedded pure pigment, mean LIR values, 2 SD, and MSWD are reported in Table 3; PDLW2-Oil corresponding to analysis of the mock-up, and PDLW2 14.7 corresponds to standard LA on the embedded pure pigment. Ellipses represent the method-specific covariance (95%). **b** Image taken with the LA system integrated microscope of the mock-up areas ablated during a session. The ablations were done to test the accuracy of the method when different ratios of pigment/oil were ablated.

**Fig. 6:**
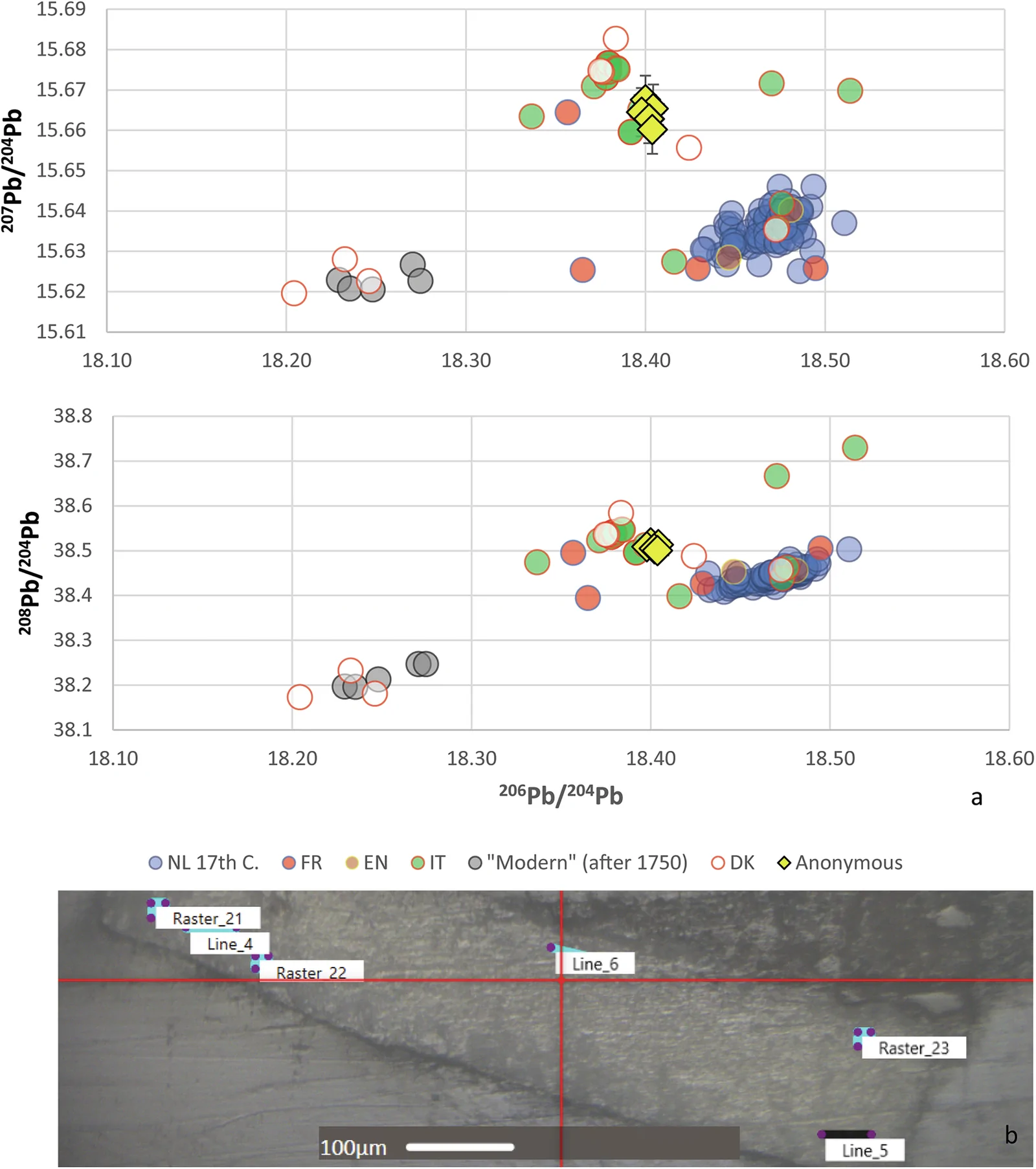
Laser ablation lead isotope ratios from an anonymous 16th–17^th^ century Italian painting are consistent with Italian lead white sources identified in the literature. **a** LIR biplots showing the results obtained for the anonymous painting compared to literature data for lead white LIR^1,2,4–7^ **b** Image taken with the LA system integrated microscope of the anonymous paint sample showing ablation location and pattern.

Figure 5a presents the LIR data for PDLW2 dispersed in oil, which exhibits a higher 2 SD compared to the oil-free reference (PDLW2 14.7). The oil-dispersed dataset splits into two distinct clusters: one consistent with the reference values obtained with standard LA on pure pigment (blue arrow) and a second group showing lower accuracy (red arrow). Visual inspection of the ablation craters (Fig. 6b) correlates these clusters with local sample heterogeneity; the accurate data (blue arrows) correspond to areas containing purer lead white agglomerates, whereas the red arrows coincide with pigment-poor, binder-rich areas. This highlights the need to consider matrix-effects when ablating oil painting samples. While signal contributions from oil-rich phases can be excluded during data reduction (e.g., via software Iolite 4; see S1 Supplementary Material Supplementary note 5*: LA in oil samples*), this significantly reduces integration time and increases measurement error. Therefore, precise selection of pigment-rich areas is critical. Ideally, LIR via LA should be preceded by SEM-EDS or FTIR imaging to guide the sampling strategy, ensuring that isotopic data is gathered from optimal locations and can be correlated with the surrounding chemical composition. The application of this method, however, shows that accurate and precise analysis of lead white in linseed oil is possible and accurate when on lead-rich areas.

### Application on Unknown 16–17^th^ century painting

The LA method was applied to a real painting sample (Fig. 6a, b), from an unknown artist, identified as probably made in Italy in the 16–17^th^ century. The sample was an embedded cross-section presenting a thick layer of lead white with small particles evenly diffused in the oil (Fig. 6b). The analysis was made using the setting with 14.7 and using an ablation time of 50 second ca., using raster and line ablation patterns and compared with literature data (Figs. 6a, b).

The LA ablation analysis of the real painting sample showed a good repeatability regardless of the sampling geometry and position in the white layer (2 SD on ^206^Pb/^204^Pb 0.0049, ^207^Pb/^204^Pb 0.0051 and ^208^Pb/^204^Pb 0.0127, data in supplementary excel file Data S1, sheet *anonymous*) and in line for what was found in this study for the application of LA ablation on fine pigment in oil paint. From Fig. 6a it is also notable that the error (to be conservative, the 2 SD used was the one calculated for oil samples analysed with LA in section Mock-up analysis, Fig. 5a and Table 3, shown as a cross on the markers of the anonymous sample) is often smaller than the marker itself, meaning that this resolution is more than enough to distinguish macro-differences between the main LIR cluster in Europe.

Data compared to the LW database for paintings show that the sample has LIR characteristics of the Italian lead white cluster, as shown in literature^1,4–8^, which is distinct compared to the lead found in other regions of Europe. This is evidence that the sample is coming from a painting that can be associated to an Italian painting made in the 16-17^th^ century.

### Mapping

**Fig. 7:**
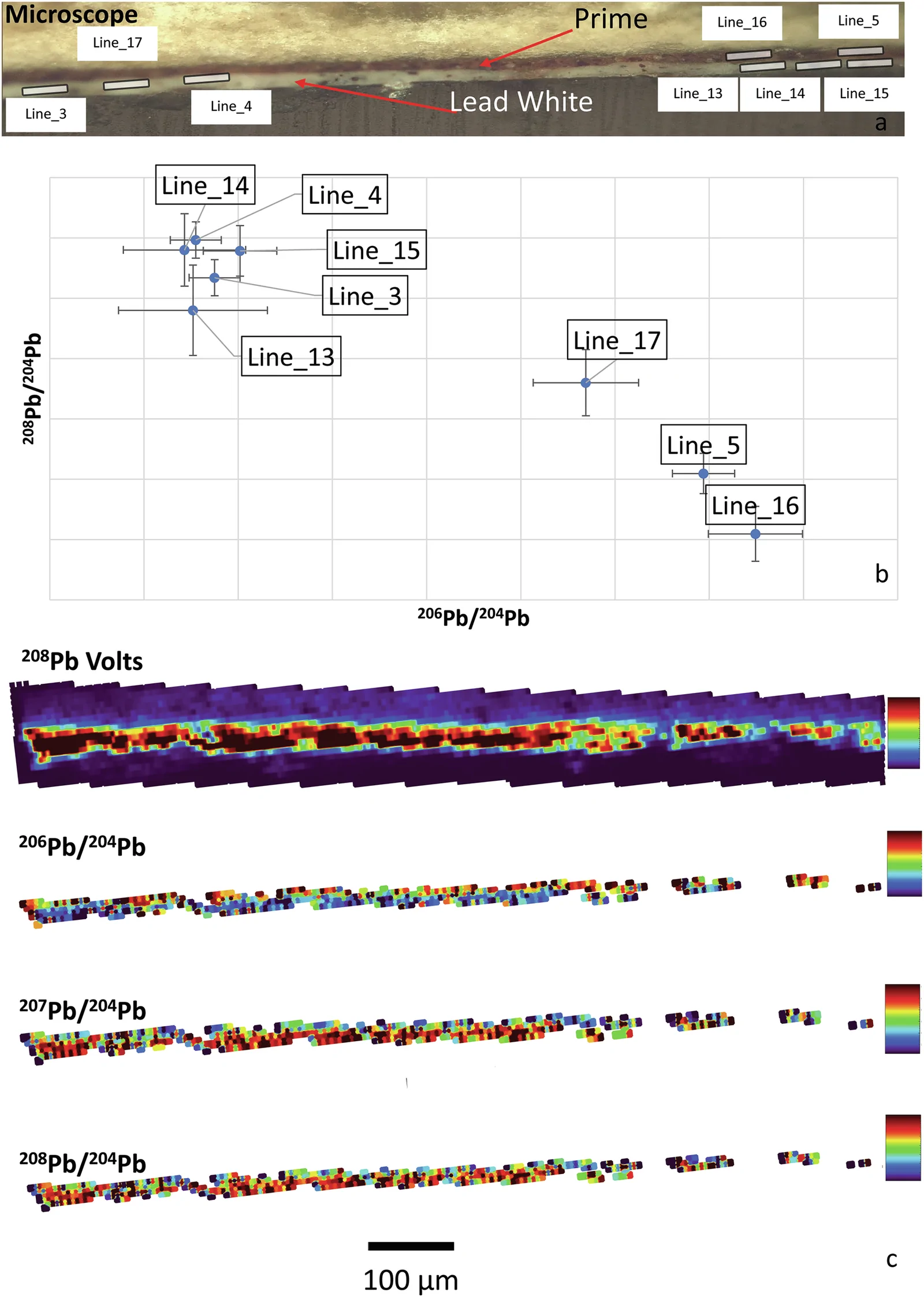
Laser ablation isotope mapping of a multilayer paint cross-section identifies two distinct lead white pigments in separate paint layers. **a** Microscope image of the cross-section showing the two different painting layers and the ablated area; **b** LIR data obtained using the LA14.7, the analyses were able to identify the presence of two different LW; **c** Mapping of the lead cross-section showing the presence of two layers of paint containing two different types of lead white.

LA systems allow the creation of elemental and isotopic mapping of the painting cross-section. While in most of the cases this is not necessary, it can be useful in certain cases, such as when thin paint layers of lead white are adjacent. This was the case for the sample provided by ARTDETECT in collaboration with the National Gallery, Alexandros Soutsos Museum, which kindly consented to share the results on the mapping (not the name of the painting and the values of the analysis, which will be discussed in a specific publication). This cross-section contains LW in two adjacent paint layers: a layer of prime (thickness estimated around 5–8 µm) and a layer of lead white with the same thickness, Fig. 7a. An initial investigation was carried out to test if the LW used in the two layers was different, using the LA14.7 settings, sampling a line of 50 µm on both layers and sampling multiple spots. The results identified the presence of two different lead whites, but a problem was encountered with lines_3, 4 and 17. While the sampling spots were on the same lead white-rich layer next to each other (Fig. 7a), their LIR were different, with line_17 showing values comparable to those obtained from line_5 and line_16 from the prime layer. After visual inspection, it was found that the surface of the cross-section was not perfectly smooth but presented protrusions and pits, which likely caused the laser sampling to be inaccurate and out of focus. Sampling in the range of 5 µm is a challenging task, as the focus of the laser ablation system camera and the laser optics is not optimal at this scale; when combined with a poorly polished surface, this can result in partial or total sampling of adjacent layers, as observed here. This explains the anomalous results for line_17, where the laser sampled the prime layer rather than the lead white layer, as visible from the LIR in Fig. 7b, where the values for this ablation fall between those of lines 3, 4, 13, 14, 15 (the lead white layer) and lines 5 and 16 (the prime).

To ensure that the two layers were isotopically distinct, that there was not a complex mixed phase of the two LWs, and to overcome the surface-related sampling limitations, it was decided to perform an isotopic mapping. The mapping provides a spatial visualisation of the Pb isotopic distribution across the sample, allowing the isotopic signature of each layer to be resolved independently of minor surface irregularities. The method development is described in S1 Supplementary material, Supplementary note 6*: Mock-up mapping*. As can be seen in Fig. 7c, with the map of the three LIRs on ²⁰⁴Pb, and especially on the ²⁰⁶Pb/²⁰⁴Pb one, the presence of two LW in distinct paint layers is clearly observed: one with lower LIR (blue pixels) and one with higher LIR (red-yellow pixels). Combining this information, it is therefore possible to state that the LW used in the prime differs from that used in the lead white layer. More broadly, this approach demonstrates how isotopic mapping can be a valuable tool for future studies, enabling the visualisation of LIR distribution across a sample and the identification of mixing or diffusion between adjacent layers that would remain undetected by conventional spot or line analysis. However, due to the nature of the settings used to generate the mapping, this method is less accurate and precise than the line analysis, and provides mostly qualitative data to understand the difference in composition within a single sample.

## Discussion

The study successfully shows that, when calibrated with synthetic lead carbonate reference pigments (PDLW1 and PDLW2), LA-MC-ICP-MS yields accurate lead isotope ratios for lead white at micrometre-scale spatial resolution while consuming only nanogram quantities of material, therefore using ~ 500000-1000 times less sample than traditional LIR analysis, making the approach micro-invasive and compatible with the practical constraints of cultural heritage sampling. The method does not require a new sample as it can operate directly on cross-sections, ablating only around 5 µm in depth.

LA results are in agreement with solution MC-ICP-MS and demonstrate that the method can produce reliable isotope ratios in a carbonate matrix without measurable bias under the operating conditions tested; the principal limitation is that external precision remains poorer than solution analysis on the same instrument. This, however, still provides the resolution typically needed to provenance of pigments. Re-analysis of historical lead white (GN) confirms accuracy on complex heritage material and comparability with prior datasets, while also highlighting a key caveat: alteration layers, residual impurities and micro-scale heterogeneity can increase measurement uncertainties, requiring careful targeting.

The mock-up cross-section experiments show that oil-rich matrices can present an analytical challenge for LA on lead carbonate: LIR precision and accuracy are worsened when ablating binder-rich, pigment-poor areas. At the same time, the results also demonstrate the method’s practical success in realistic conditions: accurate LIR are obtained when pigment-rich agglomerates are targeted, and oil contributions can be screened during data reduction, albeit at the cost of shorter usable integration time and therefore larger uncertainty. A key limitation in oil paints is that analytical performance becomes strongly dependent on micro-texture and pigment concentration; consequently, a robust workflow should include prior micro-analytical characterisation (e.g., SEM-EDS and/or FTIR imaging) to optimize ablation placement and to contextualize intra-layer variability. At the same time, this requirement underscores a major advantage of in-situ LA-based LIR analysis: it can be integrated with complementary micro-scale techniques on the same cross-section, enabling correlative, stratigraphy-resolved datasets that may provide new constraints on material choices, paint preparation, and workshop practice.

The application to an anonymous 16–17^th^ century paint cross-section further supports the feasibility of this method to real samples: the lead white layer produced internally consistent LIR across different ablation geometries and positions, and the resulting isotopic signature is consistent with Venetian LW found in Italy during that period.

Isotopic mapping demonstrates that LA-MC-ICP-MS can generate spatially resolved LIR images that identify LIR differences between layers containing different types of lead white. This is a powerful tool that can be used in order to detect the presence of different LW in different layers, as ground, prime and paint layers, as shown in the application of the method to a real sample, which identified the presence of different types of LW in two thin adjacent paint layers (~8 µm thickness). Moreover, this approach can be used in future studies to better understand and track the migration of lead species in certain mechanisms, such as the formation of lead soap.

Overall, the method provides an effective route to minimally invasive, spatially resolved LIR analysis of lead white in paintings and the workflow can be transferable to other media. In particular, it should be evaluated on frescoes and mural paintings where pigment presence is high. Further work should focus on strengthening the validation of matrix-matched standards and independent comparison with established reference approaches (e.g., double-spike TIMS), together with broader inter-session datasets to refine external reproducibility and uncertainty models. The use of new laser ablation systems, such as femtosecond pulsed lasers, should also be addressed in future applications to verify if the use of shorter pulse duration can limit matrix effects, thereby improving accuracy and precision.

## Supplementary information

Supplementary Information

Rcodes

Supplementary Data 1

Supplementary Data 2

## Data Availability

The data that support the findings of this study are available from the supplementary material DataS1 and DataS2.

## Code availability

The R codes used to generate the graphs in Figs. 2–5 are available as supplementary material as zip file Rcodes.
