# Supplementary material for: In-situ laser ablation MC-ICP-MS of lead white in paintings
Source: NPJ Herit Sci. 2026 Jun 13;14(1):513. doi: 10.1038/s40494-026-02720-3 (PMC13623631; doi:10.1038/s40494-026-02720-3)
Supplement: Supplementary file 1 [file 40494_2026_2720_MOESM1_ESM.pdf]

- Supplementary note 1: metal LIR vs LW LIR (end saying PDLW1 std 2 as quality)

Figure S1 compares the analysis of the two lead white standards (PDLW1 and PDLW2) in both their metallic and pigment forms. Each metallic coil was sampled eight times at positions evenly distributed along its length, from both the front and rear sides, to assess isotopic homogeneity of the metal. As shown in Figure S1 (PDLW1-metal and PDLW2-metal), both metals exhibit homogeneous LIR, with 2SD values comparable to those obtained for the solution analyses of the in-house standard CPI reported in Table 2.

The powdered pigments were ground and homogenised prior to analysis, and eight aliquots of each standard were dissolved and analysed by MC-ICP-MS Neoma (n = 40) over a period of six months. Figure 2 shows that the isotopic composition of each lead white pigment is indistinguishable from that of the corresponding metallic precursor, indicating that no measurable isotopic fractionation occurs during lead white synthesis, and that their 2SD values are comparable to those of the CPI in-house standard. The two resulting standards, PDLW1 and PDLW2, also display a clear difference in LIR (1229 ppm for $^{206}Pb/^{204}Pb$, 238 ppm for $^{207}Pb/^{204}Pb$ and 671 ppm for $^{208}Pb/^{204}Pb$). This difference is well suited to our purposes: it is smaller than the typical isotopic offset between Italian and Dutch lead whites, yet large enough to provide a stringent test of the efficiency of the LA method for determining LW LIR in the mock-up samples and to evaluate how these data can be applied in provenance studies.

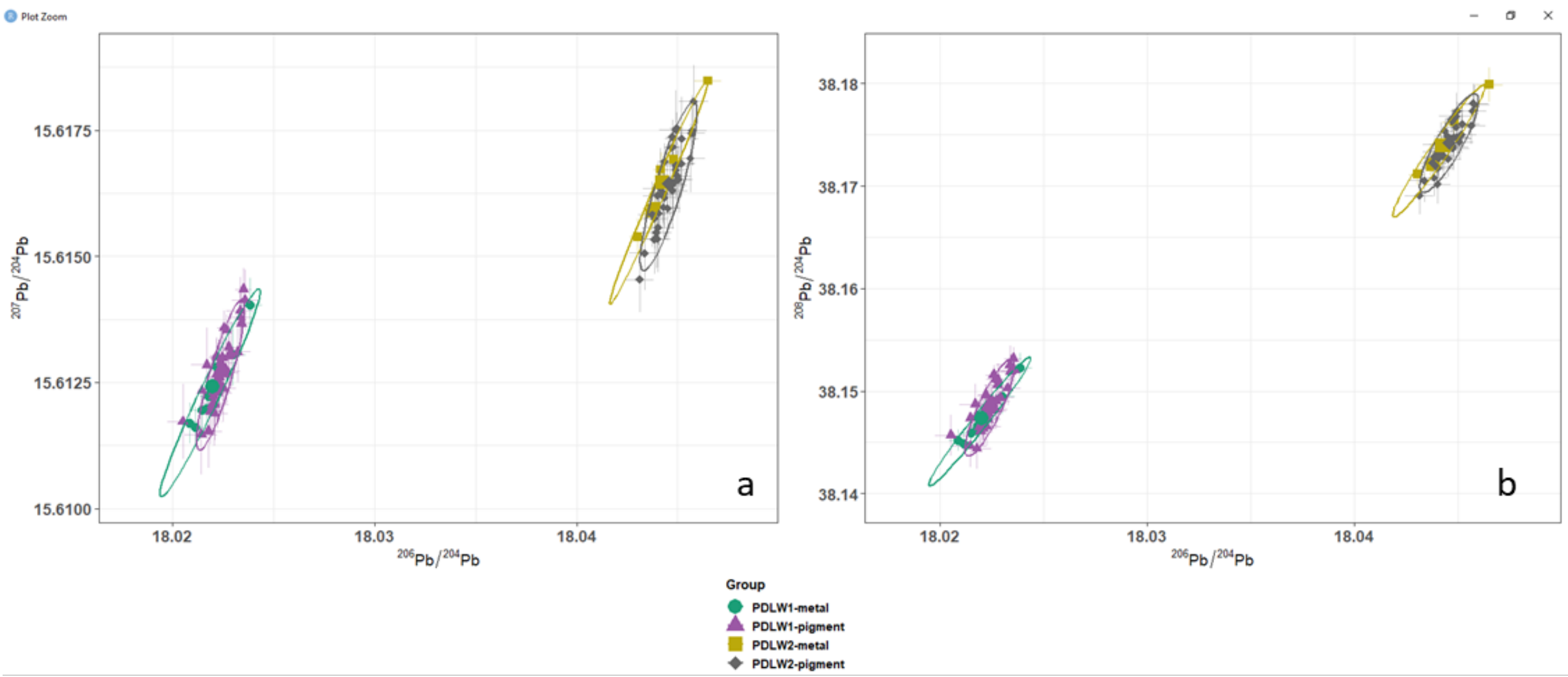

Group	206Pb/204Pb	207Pb/204Pb	208Pb/204Pb
PDLW1-metal	18.02199 ±0.00199 (MSWD=3.62)	15.61242 ±0.00165 (MSWD=2.85)	38.14735 ±0.00504 (MSWD=3.62)
PDLW1-pigment	18.02238 ±0.00123 (MSWD=0.88)	15.61271 ±0.00138 (MSWD=1.49)	38.1486 ±0.00434 (MSWD=1.97)
PDLW2-metal	18.04424 ±0.00209 (MSWD=3.59)	15.61646 ±0.00191 (MSWD=3.62)	38.17393 ±0.0058 (MSWD=4.36)
PDLW2-pigment	18.04457 ±0.00125 (MSWD=0.85)	15.61644 ±0.00152 (MSWD=1.68)	38.17422 ±0.00433 (MSWD=1.8)

Figure S1: Comparison between the analyses of PDLW1 and PDLW2 in their metal and carbonate forms, shows that there is no difference between the LIR in the two forms and that the two pigments show significantly different LIR. (a) biplot with $^{206}Pb/^{204}Pb$ vs $^{207}Pb/^{204}Pb$; (b) biplot with $^{206}Pb/^{204}Pb$ vs $^{208}Pb/^{204}Pb$. Data are available in DataS1

- Supplementary Note 2: Short discussion on the comparison with the Neptune

Table S1 reports the values for the in-house standard CPI analysed during the course of this project (6-month c.a. for a total of 203 replicates of the solution). The data are compared against the value

obtained for analysing the same solution on the MC-ICP-MS Neptune using standard sample bracketing (SSB), used for the analysis of lead white in [1–5], and the value obtained analysing the same sample in Klaver et al 2015, that characterised CPI using double spike (DS) technique on three replicates.

The data of the three techniques are within analytical error for the $^{206}Pb/^{204}Pb$, $^{207}Pb/^{204}Pb$ and $^{208}Pb/^{204}Pb$. The external error (2SD) of the Neoma MC-ICP-MS is lower compared to the one obtained on the Neptune. Note that the total number of replicates and time span for the Neoma is inferior, therefore the 2SD for the Neoma could change in the future as more analyses are conducted. The DS analyses were done only in one session using 3 replicates, therefore there is no possible to compare the external error on the CPI of the DS TIMS analysis vs the Neoma Tl doped analysis. There is a bias between the values obtained via the three methods, between 50 and 150 ppm for the three ratios. This bias is not significant as within error, and consistent with data reported in literature [6]. For the present project, it doesn't affect the provenance study of lead white. More tests will be done in future to understand the difference between these data, especially characterising better the standards PDLW1 and PDLW2 using DS technique, and evaluate if correcting for it.

		$^{206}Pb/^{204}Pb$	2SD	$^{207}Pb/^{204}Pb$	2SD	$^{208}Pb/^{204}Pb$	2SD	$^{208}Pb/^{206}Pb$	2SD	$^{207}Pb/^{206}Pb$	2SD
**Neoma n = 203**	CPI in-house Tl	17.9006	0.0015	15.5665	0.0020	38.0230	0.0060	2.12409	0.00018	0.86961	0.00005
	ppm		85.1		129.8		159.0		83.5		58.0
**Neptune n > 500**	CPI in-house SSB	17.8994	0.0031	15.5656	0.0034	38.0186	0.0113	2.12398	0.00033	0.86962	0.00009
	ppm		171.2		220.9		296.3		155.7		102.7
**TIMS n = 3**	CPI in-house DS	17.8996	0.0002	15.5645	0.0003	38.0177	0.0009	n.a	n.a	n.a	n.a

Table S1: LIR results on the in-house standard CPI with error reposted as 2D (both absolute and in ppm)

- Supplementary Note 3: Decision on the optimal laser ablation settings for lead white:

During the optimisation of the laser ablation analysis of lead white, several laser settings were taken into account. Moreover, two different ablation methods were studied: spot analysis or line/grid analysis.

Grid/line	Area (μm²)	speed μm/sec	spot μm	Energy %	Energy (J)	Hz	Integration time	Ablation time sec	N (dosage)	N*Energy
	225	4	5	1	0.42	10	0.1	12	12.50	5.25
	225	2	5	1	0.42	8	0.1	25	20.00	8.40
	225	2	5	5	0.51	8	0.25	21	20.00	10.20
	225	1	5	1	0.42	7	0.5	52	35.00	14.70
	225	1	5	1	0.51	7	0.5	16	35.00	26.95
	400	1	5	10	0.77	10	0.5	70	50.00	38.50
	225	1	5	10	0.77	10	0.5	50	50.00	38.50
	400	1	4	20	2.14	5	1	85	20.00	42.80
	400	0.7	4	20	2.14	5	1	120	28.57	61.14
	400	1	2	40	6.25	5	1	78	10.00	62.50
	400	0.7	2	40	6.25	5	1	110	14.29	89.29

Spot										
	176.7	0	15	5	0.51	2	1	113	226.00	115.26
	78.5	0	10	20	2.14	2	1	111	222.00	475.08

Table S2: Setting used to ablate lead white pigments PDLW1 and PDLW2 (Supplementary material S5).

Each type of setting was tested several times in the course of different sessions over different weeks. For the grid/line parameters, the area is calculated taking into account a square (15x15 micrometres for 225 and 20x20 micrometres for 400). Later in the study, the 20x20 approach was abandoned as it was believed to be too invasive and covering an unrealistic too large area, as pure particles of LW of that dimension do not occur commonly in paintings.

For each set of parameters, the Dosage and the product of the dosage for the energy output of the laser were calculated, and used to estimate the best settings.

To identify the best settings, the accuracy (compared to the solution analysis of the pigment reported in this study) and precision (in ppm) for the three lead isotope ratios $^{206}Pb/^{204}Pb$, $^{207}Pb/^{204}Pb$ and $^{208}Pb/^{204}Pb$ were plotted against the N*Energy (total energy delivered per area), Figure S2. The results were also reported in a biplot graph for the isotope ratios $^{206}Pb/^{204}Pb$ vs $^{207}Pb/^{204}Pb$ and $^{206}Pb/^{204}Pb$ vs $^{208}Pb/^{204}Pb$ in Figure S3, with 2SD, average and MSDW for each group of settings.

The best results for accuracy and precision were found for Values of N*Energy between 10-20, corresponding to dosages between 20 and 35. The groups at 10.2, 14.7 are the ones giving more consistent and accurate data compared to the solution work of the same pigment PDLW2 (2SD comparable to the solution work presented in D'Imporzano et al 2021). For the laser ablation analyses (n ≈ 20) of the three Pb isotope ratios normalised to ^{204}Pb yielded 2SD values of 150–300 ppm with MSWD between 0.5 and 1.1, while solution analyses (n ≈ 40) produced tighter 2SD of 80–150 ppm and slightly higher MSWD of 0.8–1.8. This indicates that the laser ablation measurements are statistically consistent with their stated uncertainties, with no evidence of significant micro-scale heterogeneity or laser-induced fractionation. Overall, the laser data provide reproducible Pb isotope fingerprints suitable for provenance work, albeit with roughly twofold lower precision than solution measurements, while both methods yield internally coherent results across all three ^{204}Pb-normalized ratios.

Spot analysis on ablation spots of 10 and 15 µm were also tested, using total acquisition times of 60-120 seconds and 1 second integration time on the MC-ICP-MS. These spot analyses yielded significantly poorer precision and/or reproducibility than raster or line scans. This degradation is most likely related to localised heating and crater evolution during static ablation, including re-sampling of partially ablated material, changes in focus, and particle melting and recondensation, all of which can destabilise the signal and enhance fractionation. In addition, spot ablation produces deeper craters than line or raster modes, increasing the risk of intersecting adjacent phases if lead white particles are confined to shallow domains, and resulting in greater visible damage to the cross-section—an outcome generally considered undesirable in conservation practice. For these reasons, the method was optimised around line and raster ablation geometries (Figure S4; comparison of spot analyses with the 14.7 protocol).

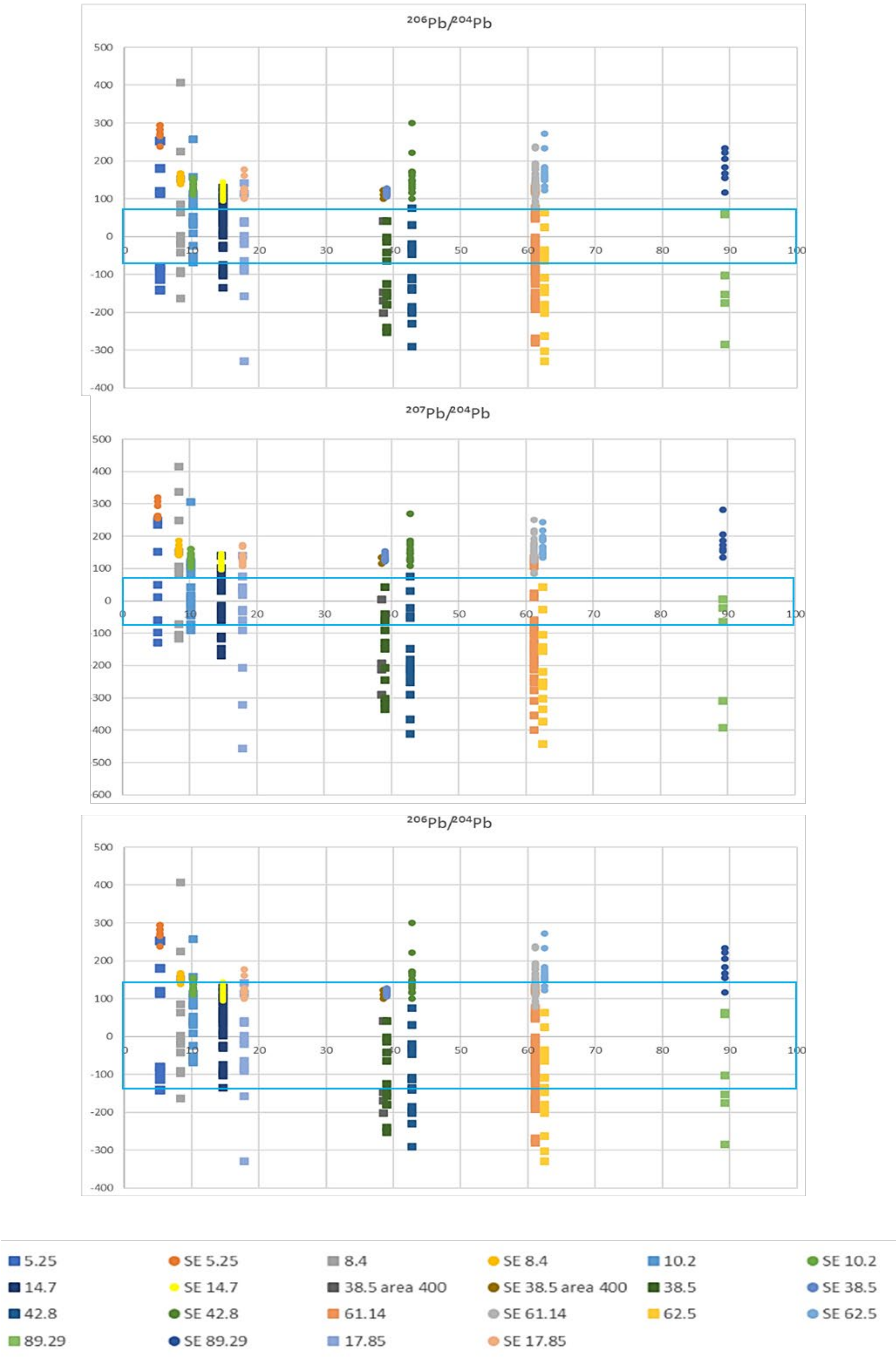

Figure S2: Accuracy and 2SE for each setting tested with the LA method on PDLW2 embedded in epoxy. The 0 on the x-axis is the value obtained by analysing PDLW2 via solution, and the blue rectangle represents the 2SD of the solution analysis on the PDLW2. Data are available in Datas2

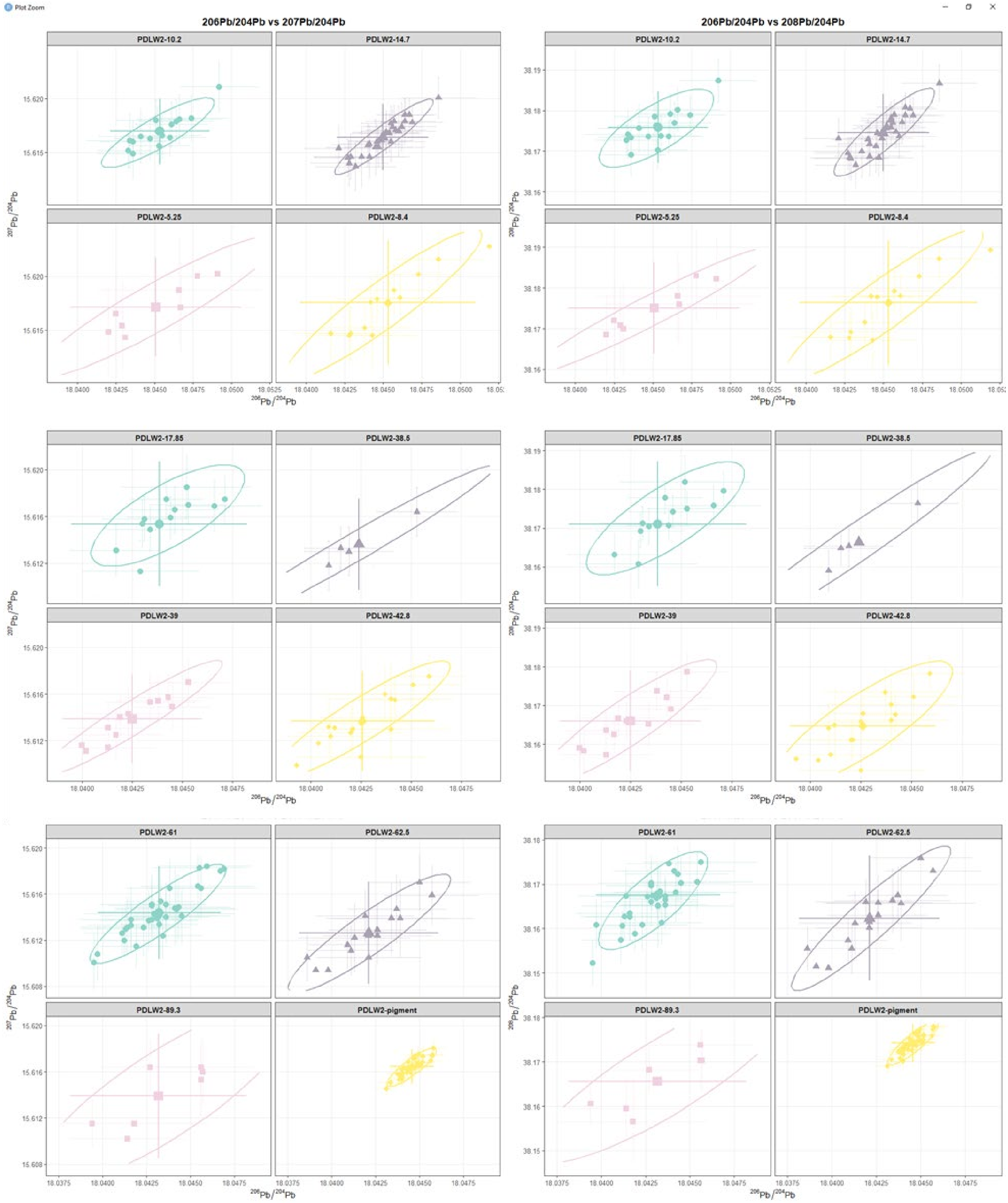
Plot Zoom
206Pb/204Pb vs 207Pb/204Pb
206Pb/204Pb vs 208Pb/204Pb
PDLW2-10.2
PDLW2-14.7
PDLW2-5.25
PDLW2-8.4
PDLW2-17.85
PDLW2-38.5
PDLW2-39
PDLW2-42.8
PDLW2-61
PDLW2-62.5
PDLW2-89.3
PDLW2-pigment
$^{206}Pb/^{204}Pb$
$^{207}Pb/^{204}Pb$
$^{208}Pb/^{204}Pb$

Group	206Pb/204Pb	207Pb/204Pb	208Pb/204Pb
PDLW2-10.2	18.04534 ±0.00322 (MSWD=0.46)	15.61697 ±0.00302 (MSWD=0.51)	38.17587 ±0.00881 (MSWD=0.78)
PDLW2-14.7	18.04498 ±0.00296 (MSWD=0.44)	15.61642 ±0.00309 (MSWD=0.67)	38.17454 ±0.00947 (MSWD=1.11)
PDLW2-5.25	18.04509 ±0.00552 (MSWD=0.32)	15.61712 ±0.0046 (MSWD=0.27)	38.17509 ±0.01122 (MSWD=0.3)
PDLW2-8.4	18.04532 ±0.00572 (MSWD=1.08)	15.61753 ±0.00582 (MSWD=1.44)	38.17638 ±0.01537 (MSWD=1.78)
PDLW2-17.85	18.04385 ±0.00438 (MSWD=1)	15.61535 ±0.00535 (MSWD=1.59)	38.17105 ±0.01613 (MSWD=2.63)
PDLW2-38.5	18.0424 ±0.00395 (MSWD=0.96)	15.61363 ±0.00392 (MSWD=0.87)	38.1664 ±0.01453 (MSWD=1.96)
PDLW2-39	18.0425 ±0.00347 (MSWD=0.7)	15.61386 ±0.00382 (MSWD=0.82)	38.16604 ±0.01305 (MSWD=1.8)
PDLW2-42.8	18.04259 ±0.0036 (MSWD=0.57)	15.61366 ±0.00427 (MSWD=0.97)	38.16481 ±0.01395 (MSWD=1.76)
PDLW2-61	18.04322 ±0.00351 (MSWD=0.45)	15.61437 ±0.00403 (MSWD=0.75)	38.16764 ±0.01338 (MSWD=1.48)
PDLW2-62.5	18.04211 ±0.00397 (MSWD=0.49)	15.61262 ±0.00442 (MSWD=0.78)	38.16231 ±0.01411 (MSWD=1.47)
PDLW2-89.3	18.04317 ±0.00501 (MSWD=0.79)	15.6139 ±0.00542 (MSWD=1.05)	38.1656 ±0.01325 (MSWD=0.96)
PDLW2-pigment	18.04457 ±0.00125 (MSWD=0.85)	15.61644 ±0.00152 (MSWD=1.68)	38.17422 ±0.00433 (MSWD=1.8)

Figure S3: Summary of laser operating conditions and corresponding analytical performance for PDLW2, including mean LIR values, 2SD, and MSWD. PDLW2-pigment represents the analysis of the pigment done in solution and taken as true values of the PDLW2. Ellipses represent the method-specific covariance (95%). Solution measurements show lower 2SD, while all three datasets agree within uncertainty. Data are available in Datas2

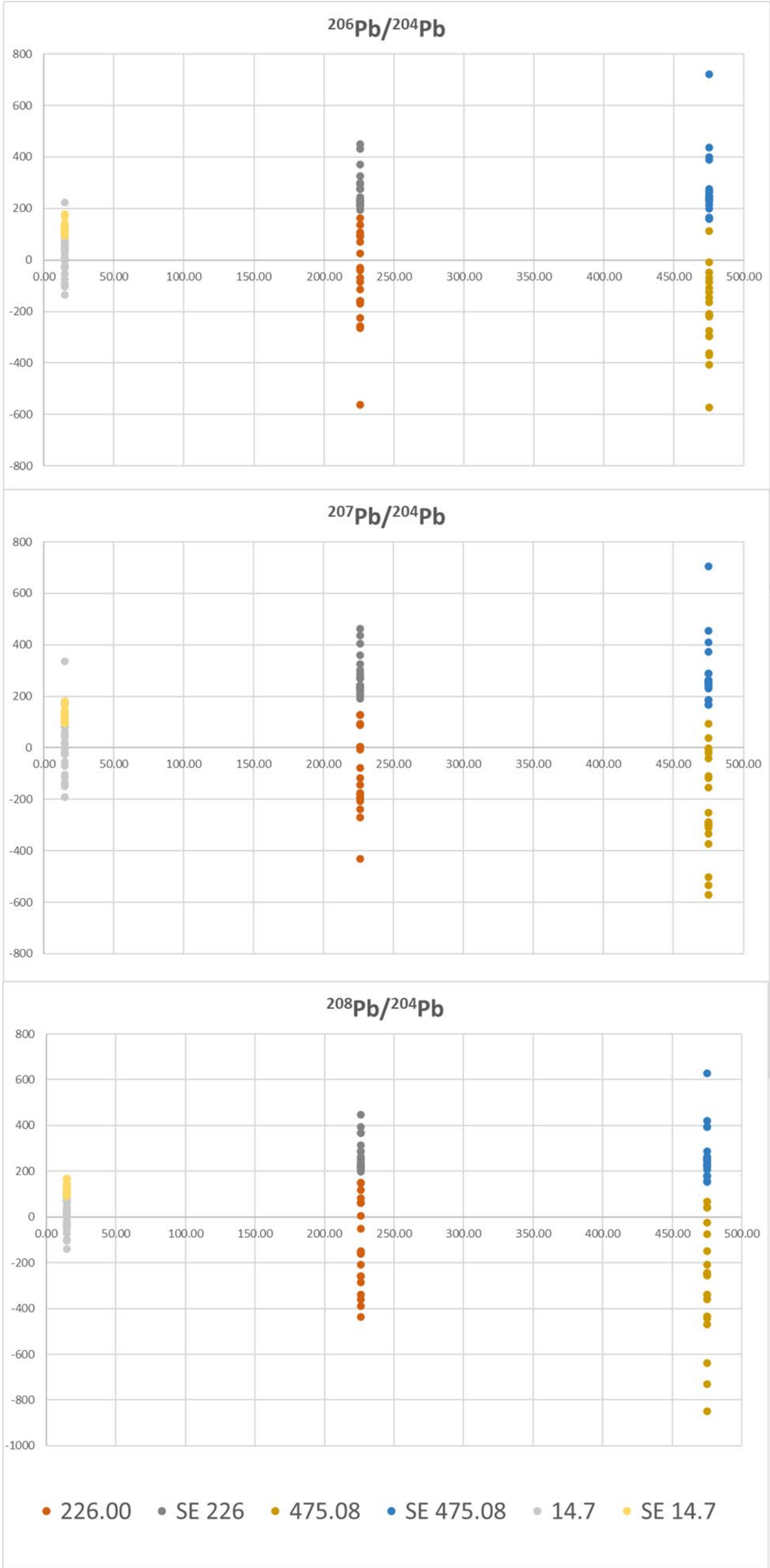

Figure S4: The figure shows the difference in precision and accuracy (in ppm) between LA using a moving beam and spot analysis. Accuracy and 2SE for spot analysis setting tested with the LA method

on PDLW2 embedded in epoxy and compared to the 14.7 LA settings. The 0 on the x axis is the value obtained analysing PDLW2 via solution. Data are available in Datas2

- Supplementary note 4: Material ablated and efficiency:

The various LA parameters were evaluated using an Alpha-Step D-500 surface profilometer (KLA-Tencor stylus profilometer) to estimate the ablation depth produced under different conditions (Figure S5). Initial tests were carried out on LA spots using a 5 µm spot size, stage speeds of 1 and 2 µm/s, and laser energies of 0.42, 0.51, 0.77, and 2.14 J, each at dosages of 35 and 20. The profilometer is equipped with a 2 µm stylus tip, which proved too large relative to the 5 µm crater diameter to allow reliable depth measurements at that spot size. To overcome this limitation, the ablation tests were repeated using a 15 µm spot with the same combinations of speed, energy, and dosage to approximate the ablation depth under the analytical conditions. Although a 15 µm crater is larger than the stylus tip spatial resolution (and experiments were not extended to larger spot sizes to avoid excessive Pb removal and potential long-lasting matrix effects in the laser system and the Neoma MC-ICP-MS), the resulting profiles provided a reasonable estimate of crater depth. In particular, the 2.14 J setting produced a partial flat-bottomed profile at 15 µm spot size, indicating that the stylus tip was able to reach and track the base of the crater. As shown in Figure S5, the ablation depth for the tested conditions lies between approximately 4 and 8 µm, and these values are used to calculate the ablated volume for the efficiency assessment.

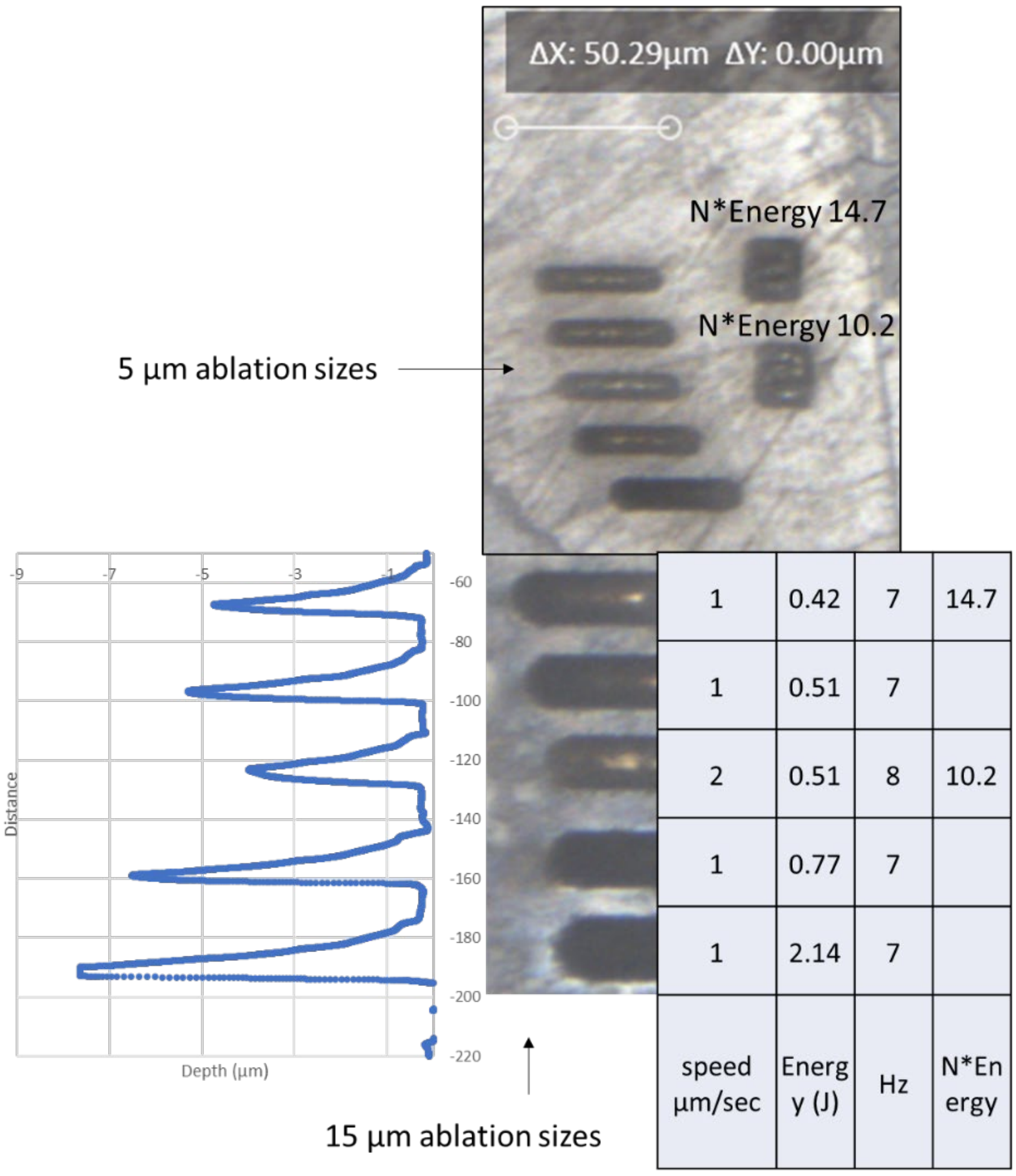

speed µm/sec	Energy (J)	Hz	N*Energy
1	0.42	7	14.7
1	0.51	7	
2	0.51	8	10.2
1	0.77	7	
1	2.14	7	

Figure S5: Optical micrographs acquired with the microscope of the laser ablation system, showing the ablation craters produced in line and raster scanning modes. The graph (bottom left) reports the

depth profiles of the different ablation conditions tested in this study, and the table lists the laser parameters (scan speed, energy, and repetition rate in Hz) used for each ablation.

Using these values, it was possible to estimate the efficiency for ablating the lead white with N*Energy of 14.7 and 10.2 was around 0.05-0.25%. This range of efficiency is a function of day-to-day tuning conditions and rheology of the carbonate during analysis.

Considering an average ablation depth of 5 µm it is possible to calculate the average amount of pigment ablated (considering a density of lead white of 6.7 g/cm^{-3} and an average length of ablation of 50 µm) around of 8.375 ng, which decreases to 3.35 ng for smaller analyses with 20 µm length ablation areas.

Laser ablation: raster vs Line

Painting samples stored as cross sections have different layers that vary in thickness and contain irregular particles of pigments. To optimise the sampling procedure for a wider variety of sample specimens, two different approaches were tested: ablating following a raster (grid) or a line. The first approach is more suitable for rounded lead white particles with a diameter of around 10-15 micrometres, while the second can be applied for sampling having a thin layer of lead white homogenously dispersed in oil paint (ideal thickness of the layer around 6-8 micrometres minimum). The line sampling signal (expressed in voltage for each isotope) is more stable than grid sampling. Sampling carbonates results in signal intensity variation of 10-20% depending on the rheology of the sample. Moreover, the raster sampling can have even more variable signal, as the trajectory of the ablation is not linear, but it has 90 degrees turn, that can cause over-ablation of the same area resulting in signal drops (as shown in Figure S4 on the ^{208}Pb voltage signal, which shows two drops in relation to the raster turning). To evaluate whether these transient intensity affects isotope ratios, raster and line scans were directly compared in terms of both signal intensity and LIR. As shown in Figure S6, the raster turning-related signal drops do not measurably affect the calculated isotopic composition. The raster/line comparison supports the use of both geometries, which can be used according to the appearance of the sample.

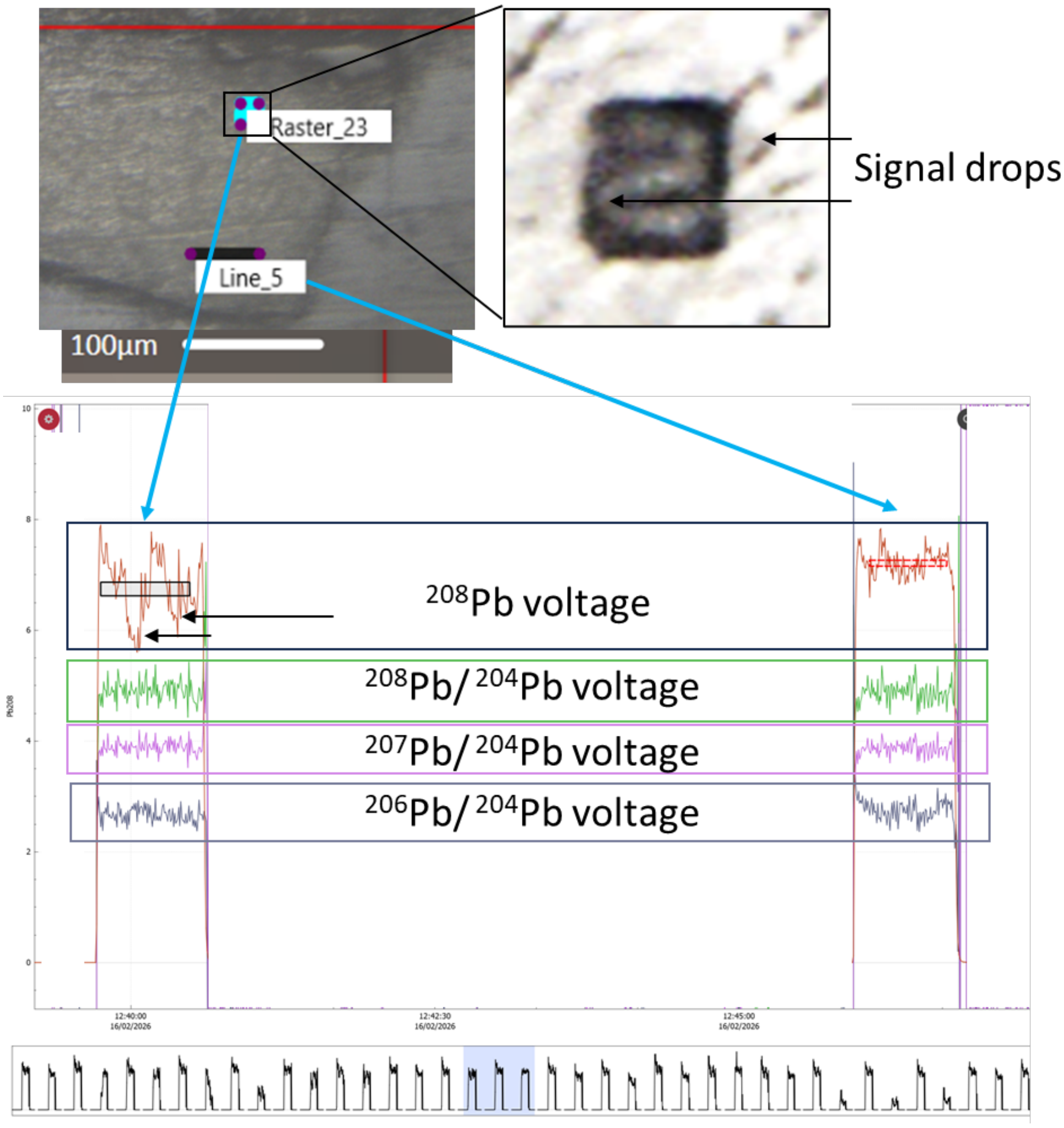

Figure S6: Voltage and LIR signal of the LA showing the differences between raster and line ablation. It is visible that while the Voltage in the raster analysis drops during the turnings, this doesn't affect the Isotopic composition.

- Supplementary note 5: LA in oil samples

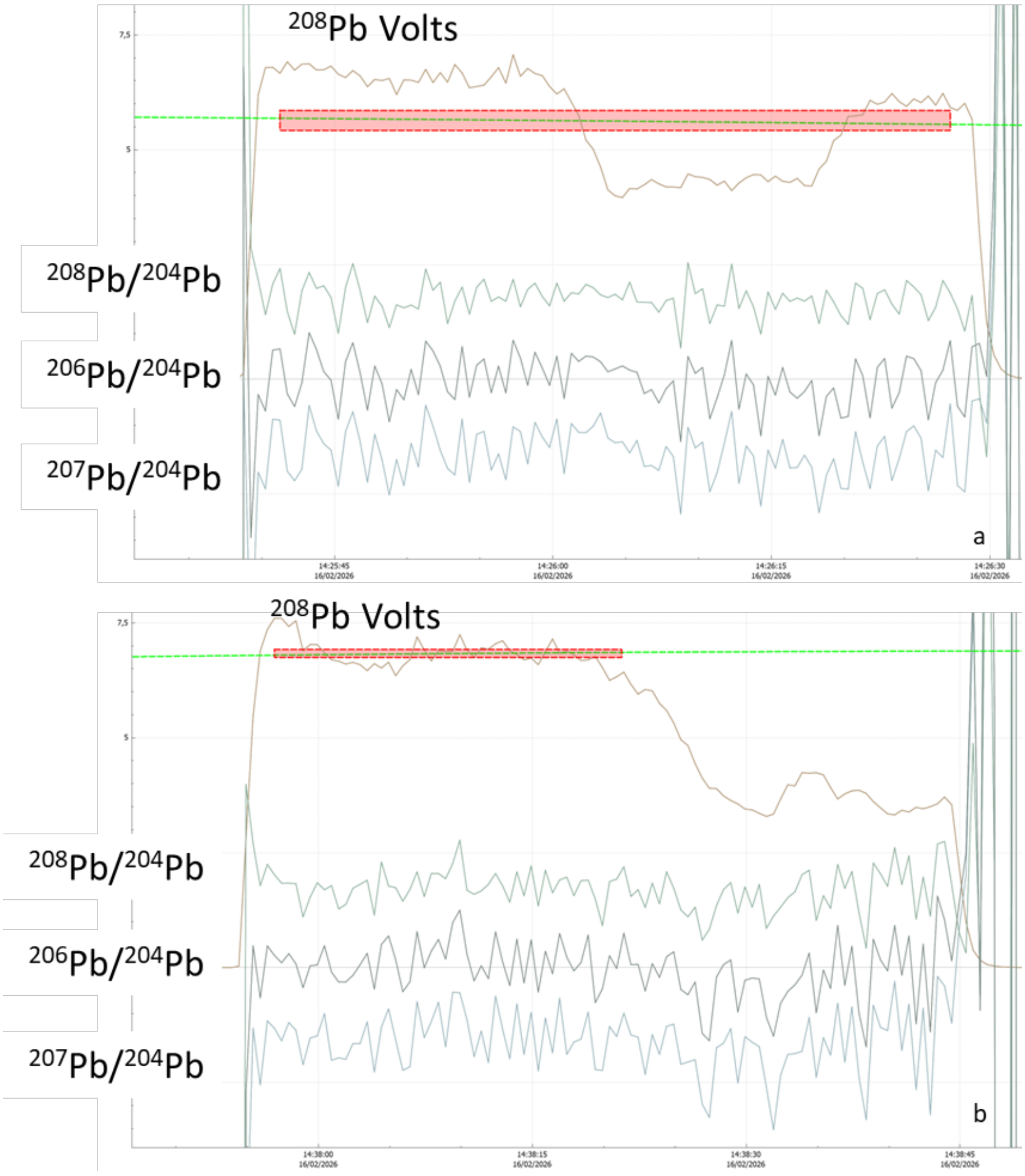

Figure S7: Volt and LIR signal of the analysis of a line in the layer containing PDLW2 in oil in the mock-up sample. The picture shows the difference and alteration of LIR if areas containing high amount of Oil are analysed. The data were generated via Iolite 4.

Figure S7 shows two examples of time-resolved signals from the mock-up, processed in Iolite, where the ablation path crosses from a lead white particle into an area with more oil. A clear drop in the ^{208}Pb intensity marks this transition. This change in matrix composition also affects the measured isotope ratios, likely due to increased fractionation in the oil-rich phase.

The impact varies between spots: in Figure S7a, the LIR remains relatively stable across both phases, allowing the entire signal (dotted rectangle) to be used. However, in Figure S7b, the ratios drift significantly in the second, oil-rich part of the signal, resulting in poorer accuracy. In such cases, it is crucial to select only the first part of the signal, corresponding to the pure lead white, for the final calculation to obtain reliable results."

- Supplementary note 6: Mock-up mapping

Figure S8 shows the LIR mapping in false colours of the mock-up sample. The analysis was made using laser settings calculated specifically for mapping purposes, obtained using a dedicated Iolite software provided by ESL laser (iolite_optimizer_v0.96). A special designed ESL plasma torch (having a smaller inner tube diameter for sample injection to reduce washout time) was used to improve the washout time. The imaging was performed with raster using a 3 µm spot size (0.5 µm separation between lines to avoid resampling), 20 Hz repetition rate, and 20 µm/s scan speed over a 1 mm × 0.3 mm area. The depth of imaging analysis is calculated to be less than the one presented in the study before (< 5 µm as the dosage used for the mapping is 3, lower than the one used for raster or line analysis), and the surface ablated can be easily polished. Data reduction was carried out using Iolite, applying a 5 V threshold to the ^{208}Pb signal when the LIR imaging was calculated. This cutoff was essential to mask areas with a higher proportion of matrix to Pb white and to use data more closely signal-matched because of the SSB approach.

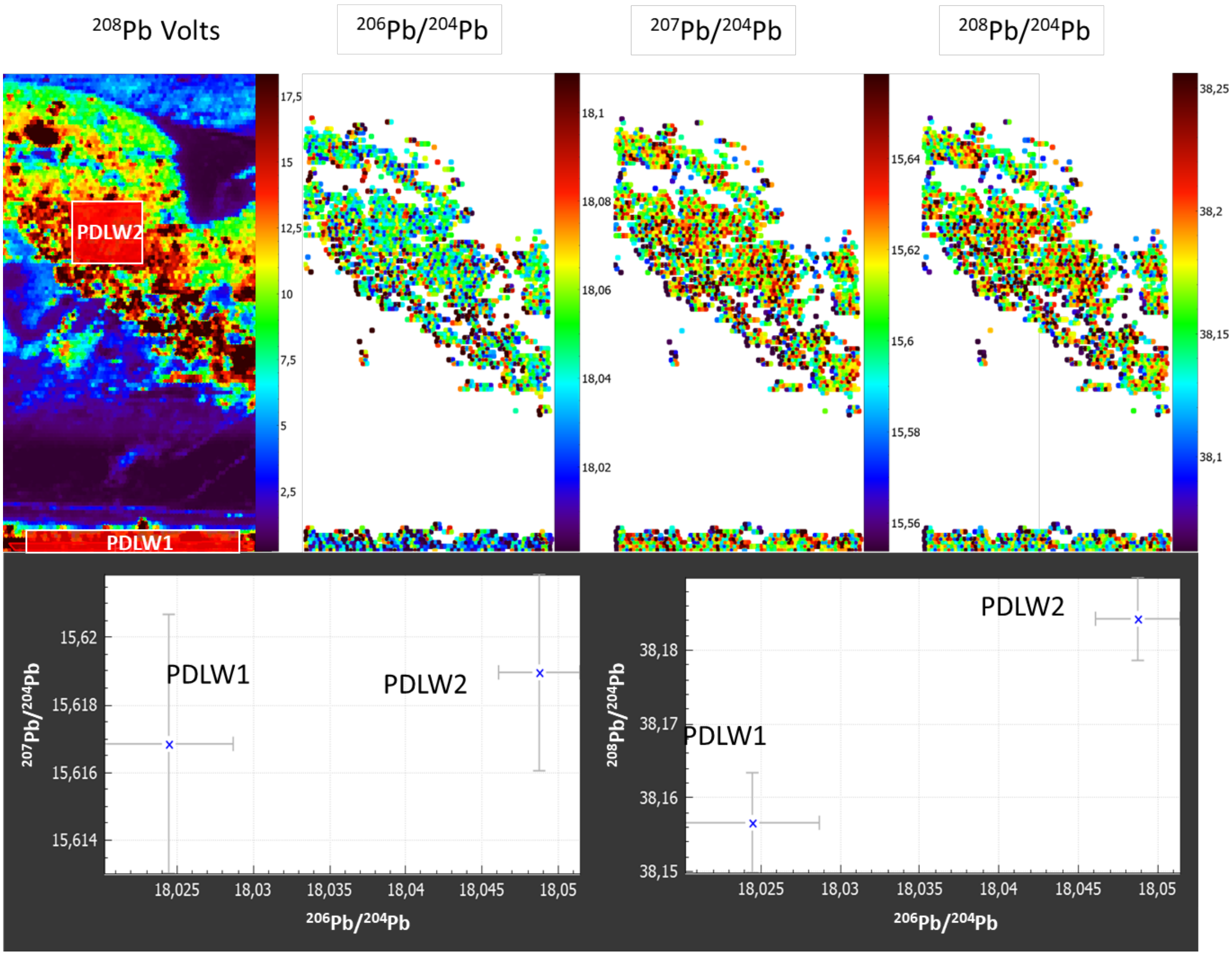

Figure S8**:** False-colour maps of the ^{208}Pb signal intensity (V) and the corresponding lead isotope ratios (LIR) calculated over the same mapped area. LIR pixels were computed only where the ^{208}Pb signal exceeded a 5 V threshold. In the 208Pb intensity map, two regions of interest are highlighted in red, corresponding to PDLW1 (lower layer) and PDLW2 (upper layer). Mean LIR values extracted from these regions are shown in the biplots below: the mapped layer averages are consistent with the bulk LIR signatures of PDLW1 and PDLW2, albeit with larger uncertainties. The data were generated via Iolite 4.

The ^{208}Pb distribution map effectively visualises the stratigraphy, with high-intensity (red-yellow) zones corresponding to lead white layers and low-intensity (blue) regions indicating zinc white. Some mechanical mixing of PDLW2 into the adjacent zinc layer is evident at the interface. Although the two lead pigments appear visually similar in the three LIR over ^{204}Pb ratios maps, the $^{206}Pb/^{204}Pb$ ratio map highlights their isotopic differences more clearly, with PDLW1 showing distinctively lower ratios (blue pixels) compared to PDLW2. Averaging isotopic ratios from two regions-of-interest in the lead-rich areas confirms that accurate LIR values can be extracted from these maps (the areas are the red rectangles highlighted in the ^{208}Pb Volts map in Figure S8).

Supplementary data:

Data S1. Pb-isotope ratios for method validation on metal, pigment and reference materials. Lead-isotope ratios ($^{206}Pb/^{204}Pb$, $^{207}Pb/^{204}Pb$, $^{208}Pb/^{204}Pb$, $^{208}Pb/^{206}Pb$ and $^{207}Pb/^{206}Pb$) and their standard errors (SE, within-run of each ratio) measured by MC-ICP-MS. Worksheets compare laser-ablation (LA) and solution-mode analyses of the metallic and pigment fractions of samples PDLW1 and PDLW2, the reference material GN (D'Imporzano et al., 2021), measurements on oil-bearing material, and an anonymous sample (reported with its mean and 2SD). The "Solution PDLW2 and LA" worksheet additionally reports the corresponding ablation efficiency.

Data S2. Laser-ablation parameters and the resulting precision and accuracy of Pb-isotope ratios. Full set of laser-ablation operating conditions and per-analysis results underlying the parameter table and depth-profile figure. The "parameters" worksheet lists, for each ablation condition, the ablated area (μm^2), scan speed ($\mu m\ s^{-1}$), spot size (µm), energy (as % and in J), repetition rate (Hz), integration time, ablation time (s), the dosage N (overlapping pulses per unit of material) and the total energy delivered per unit area. The "SE + Accuracy" worksheets report, for every individual ablation, the measured Pb-isotope ratios with their internal standard error (SE) and their accuracy expressed as the bias from the accepted reference value (with SE in ppm).

References:

1. D'imporzano P, Reiling HM, Merkel S, van Iperen JM, Garachon I, Keune K, et al. Lead isotope analysis of lead-tin-glaze via on-site portable laser ablation sampling of 17-18th century Delftware (earthenware). J Archaeol Sci. Elsevier; 2026;185:106443.

2. D'imporzano P, Batur K, Keune K, Koornneef JM, Hermens E, Noble P, et al. Lead isotope heterogeneity in lead white: From lead white raw pigment to canvas. Microchemical Journal. Elsevier; 2021;163:105897.

3. D'Imporzano P, Davies GR. Lead Isotope Ratios of Lead White: From Provenance to Authentication. In: Colombini MP, Degano I, Nevin A, editors. Analytical Chemistry for the Study of Paintings and the Detection of Forgeries [Internet]. Cham: Springer International Publishing; 2022. p. 447–71. https://doi.org/10.1007/978-3-030-86865-9_14

4. D'Imporzano P, Keune K, Koornneef JM, Hermens E, Noble P, Vandivere ALS, et al. Time-dependent variation of lead isotopes of lead white in 17th century Dutch paintings. Sci Adv. American Association for the Advancement of Science; 2021;7:eabi5905.

5. D'Imporzano P, Keune K, Koornneef JM, Hermens E, Noble P, van Zuilen K, et al. Micro-invasive method for studying lead isotopes in paintings*. Archaeometry. 2020; https://doi.org/10.1111/arcm.12549

6. Thirlwall MF. Multicollector ICP-MS analysis of Pb isotopes using a 207Pb-204Pb double spike demonstrates up to 400 ppm/amu systematic errors in Tl-normalization. Chem Geol. Elsevier; 2002;184:255–79.
